# Spirit of the English Periodicals, and Notices of English Medical Literature

**Published:** 1837-07-01

**Authors:** 


### Spirit of the British and American Periodicals, and Notices of British Medical Literature.

SINGULAR CASE OF PERIODICAL MONOMANIA, TERMINATING IN SUICIDE, WITH THE APPEARANCES ON DISSECTION. By JAMES JOHNSON, M.D.

I THINK I may venture to assert that there is not a more remarkable—perhaps a more interesting case in the annals of medicine, than the following.

Mr. M'Kerrell, aged about 56 or 7, had spent nearly thirty years in the civil service of the East India Company, and had risen to a high rank on the Madras establishment. He returned home about six or seven years ago, with an ample fortune. He was a gentleman of highly cultivated mind, and great natural abilities. He had suffered some severe attacks of fever in India; but arrived in his native land with a comparatively sound constitution. He had made some excursions to the Continent, more for amusement than health, and, about four years previous to his death, had sustained an unsuccessful contest for the representation of Paisley. In this contest he went through an immense exertion of body, and excitement of mind. He dated his chief bodily and mental afflictions from this period. His digestive organs became considerably deranged, and constipation of the bowels was a prominent feature of the malady, up to the very day of his death—which was occasioned by his own act. Very soon after the contest alluded to, he came under my care, and continued to consult me, with longer or shorter intervals, till the final and melancholy termination of his existence. At first, his corporeal ailments were alone referred to—and these were the common symptoms described by the generality of dyspeptics. There was an appearance in his manner, however, which led me to suspect some grievance kept in the back ground. The patient, being a man of great intelligence and knowledge of the world, he frequently detained me in conversations and discussions quite foreign to the objects of my visit, and dismissed me without even entering upon them at all. One day, he appeared more than usually depressed, and, after some ordinary conversation, he remarked that he thought me a man in whom he could confide, and who would not divulge a secret, if a pledge of secresy were given. I observed, a little jocularly, that doctors were like priests, and confessions were held as secret by the physicians of the body as of the mind. But I observed, at the same time, that I had no wish or curiosity to become acquainted with secrets unconnected with my professional avocations. That, said he, I believe; "but this is a secret directly connected with my health and happiness; and I think its disclosure to a man of honour, and professional experience, might probably enable him to relieve me. But I had rather suffer the miseries which I endure, than confide the CAUSE of them to a physician who would divulge it." In that case, I readily pledged myself to secrecy, provided there was nothing in the secret of a criminal nature, and which the laws of God or man did not call upon me to make known, if necessary. He answered, "if you find in this secret any thing criminal, I absolve you from your promise." I then pledged myself.

It was with great difficulty, and tedious circumlocution that he came to the point, being apparently tortured with shame in the disclosure. At length it came out that he laboured under an illusion, which came on regularly every second day, and lasted till he went to sleep. He would awake the next morning free from the hallucination, and perfectly convinced that it was an illusion under which he had laboured on the preceding day. At a later period, he often assured me that he was conscious of the illusion *being such*, and not a reality, on the days of its occurrence, though I have strong reason to suspect this statement, for, up to the day of his death, he never would see me on the day of the illusion. The illusion itself let in no light on the nature of the complaint, and therefore the secret may be kept, without any detriment to the history of this extraordinary case. Unfortunately the entity in which it consisted, presented itself so frequently, and was mixed up with so many other objects and subjects, that he could not walk a street without seeing it, unless he kept his eyes shut—he could scarcely read a page of a book without its crossing his sight—he could hardly, indeed, survey the furniture and other things in his room, without being reminded of this torturing illusion. His sufferings were of two kinds: One, directly connected with the illusion itself, which, according to his conviction, on alternate days, exercised a hostile influence on him, and was, as it were, an evil star, blasting every thing belonging to him by its malign radiation! The other, was an indescribable horror, terror, or sense of destruction, having no direct reference to the illusion, and a degree of which continued after the delusion vanished, that is to say, during the "good days." This was the part of the complaint which appeared, both to the patient and the physician, as purely corporeal, the result of some disordered function or structure of the body. The illusion, on the other hand, had greater reference to the mental functions, though probably they were both the result of the same cause.

I made numerous and minute examinations of the state of the general health, without being able to detect any palpable cause for these strange phenomena. In respect to the head, this gentleman was highly gifted in intellectual powers, and his mind amply stored with varied and extensive information. He never displayed the slightest aberration, or even weakness of mind, except as regarded the hallucination—and even *that* he regarded in its true light, when the mental cloud had passed away. There was no physical symptom of any cerebral or spinal affection. The senses and sensibility were perfect—as were all the muscular powers. He was, however, emaciated, and the emaciation gradually, though slowly increased.

I frequently examined the chest, and it sounded well, in all parts—the patient could expand it freely—and he could take great exercise without any shortness of breath. He had no cough. The pulse was remarkably small, and generally slow. I confess that, at no period of my professional attendance, which lasted three years and a half, did I suspect any disease of the heart or lungs, though in both cases, I was mistaken, as the final event proved.

In respect to the abdominal viscera, there were many symptoms of disordered digestion, but none of organic disease. He ate two meals of animal food daily, the aggregate being considerably more than a man in health, and using but moderate exercise, would naturally consume. The patient found it necessary to confine himself almost entirely to plain animal food, with bread, and very little wine, or weak brandy and water. Any deviation from this system occasioned considerable inconvenience, both as to the common symptoms of indigestion, and also as respected the main and paramount malady. The constipation of bowels, as before remarked, was very obstinate, and the fæces were generally in the form of scybala, even when aperients were employed. All strong or drastic purgatives aggravated his complaints. The functions of the kidneys were very rarely disturbed. Thus, then, repeated examinations of the various organs, through the medium of their functions, disclosed to me no tangible or cognizable proof of structural disease. Yet such disease did actually exist, however humiliating may be the confession. This does not militate against the science of medicine, but only against my own powers of discrimination.

The regular, the unerring periodicity of the main symptoms puzzled me not a little; and this periodicity I could not help attributing to some physical, rather than to a moral cause. What tended to embarrass me still more was the fact that, on the "good day," he could view the ENTITY, whether simple, or in any of its combinations, with composure, while on the "bad day," or the day on which the illusion was in the ascendant, the sufferings which this unfortunate, and highly-gifted individual experienced, were such, that the narration of them often rendered me, as it were, a participator of his miseries, and deprived me of sleep. I can conscientiously say that I heartily repented of being the depositary of the secret, and the physician of the patient.

I had ample reason to believe that he frequently sent for me, without any intention of making use of my prescriptions; but merely to hold a conversation, or rather disputation respecting his malady. His leading questions were generally whether or not an illusion of the mind could exist, independently of bodily disorder; which I always answered in the negative. One day he asked me directly, and requested a candid and unequivocal answer, whether I did not consider the illusion under which he laboured on alternate days, as a species of INSANITY? I told him that certainly it came nearer to MONOMANIA, or delusion on one particular subject, than to any other affection to which I could give a name.—After a short pause, during which considerable emotion was visible in his countenance, he remarked that I was perfectly right—and though he was exceedingly anxious to conceal his infirmity from the world, he could no longer conceal it from himself, especially as it was confirmed by me. He then, and often afterwards, observed that, when the human mind lost its poise, even on a single point, life was no longer worth preserving. He then, too, and many times subsequently, said that his mental and corporeal sufferings were such as would drive many a man to commit suicide; but that he entertained too high a religious and moral sense to destroy his own life.

One day he asked me, could a man be considered insane who was conscious that he was so? This was a puzzling question! I replied that certainly one strong feature of insanity was a firm belief, in the mind of the insane individual, that he himself was of sound mind. But I remarked that it was exceedingly difficult to form an unexceptionable definition of any disease, and more especially of insanity—and that probably the present instance might prove an exception to the general rule. I then put this home-question to him. Do you believe that you labour under monomania, or delusion on one particular subject, during the time the illusion exists;—in other words, on the "bad day?" After some hesitation, he replied that, for a long time, the illusion appeared to him to be a reality, during its domination, and the suspicion of monomania had not then entered his mind; but that, at present, he believed the illusion to be such, even when under its influence, which influence he could no more resist than he could prevent the clouds from passing over Regent-street. The same, he observed, was the case respecting his belief in the existence of monomania on the bad days:—*he did believe, even on the days of the illusion, that he laboured under partial insanity.* This, then, would appear to decide the question. But there is a link in the chain defective. He never gave me the opportunity of verifying this declaration, either as to the belief in the non-reality of the illusion, or the reality of the monomania, on the "BAD DAY." This is, in my opinion, a very important defect; and excites in my mind, a strong suspicion that, during the day of the illusion, he believed in its reality, and disbelieved his own aberration. When the hallucination was over, it is not at all improbable that his powerful intellect might come to the conclusion that, during the preceding day, his reason was obscured by the illusory phantom which had so long embittered his life.

Since the melancholy issue of the case, I have learnt from his nearest relatives, that the regularity of his periodical attacks was well known to them: so much so that, when any letter was despatched to him, they consulted the almanac, in order that it might arrive on the good day. A mistake was once made in the calculation, and very serious consequences were the result.—His friends knew nothing of the mental illusion, but only that he laboured under a periodical disorder of body which indisposed him for any business. I say *indisposed* as contradistinguished from the term *incapacitated;* for it is my firm conviction now, as it was all along, that, upon every point not directly connected with his illusion, his reasoning powers were as perfect and clear as at any period of his life. Of this, at least, I am certain, as far as the good days were concerned; and I believe that the same applied to the days of the illusion. Still I can only offer this as my belief. The integrity of his intellect on the "good days," was a matter of knowledge.

In the Spring of 1835, I saw him frequently, and he appeared rather worse than usual, there being now an additional source of misery—evidently a dread of the world's coming to a knowledge of the illusion and its cause —MONOMANIA—I firmly believe that this fear was so strong, that some of the last acts of his life, and especially the letter to his landlady, announcing his determination to commit suicide, were done with a firmness and coolness which might lead the jury to bring in a verdict of *felo de se,* instead of lunacy. So well-informed a man as he was, could hardly be ignorant that, by such a verdict, his property would be forfeited to the Crown; but this turned not the scale, when placed against the disclosure of an infirmity of mind, which the unfortunate gentleman wished so much to conceal.

In this place, it may be properly enquired whether the gentleman in question was a proper object for restraint. As far as I was concerned, I conceive that I had not the slightest cause or right to attempt any privation of his liberty. He never hinted the slightest intention to commit suicide—but the contrary; and the delusion under which he laboured, affected no man's interest, but only his own happiness. Why then should he be confined? Besides, it requires the testimony of two medical men to deprive a man of his liberty —and in this case, there was only *one* privy to the illusion, nor was it possible for any other medical man to become acquainted with it, by his own personal knowledge. Had it been proper or prudent, therefore, to confine this gentleman, it could not have been done legally. He once wrote a letter to a relation, announcing his intention of committing suicide, in the same form as his last letter was written to his landlady. He did not then put the threat into execution. But even this announcement of his intention to commit such an act, did not authorise restriction. The law does not say that suicide itself is a necessary proof of insanity; else why do we see the verdict of FELO-DE-SE occasionally brought in? And if the committal of the crime be no legal proof of insanity, the mere threat of it would be still less so. I have alluded to this question, because it was agitated in the course of the inquest, and also because some of the periodical prints (the ATLAS for example) have quoted the case as elucidating the distinction which Dr. Mayo has drawn between *moral* and *mental* insanity. I think that neither law nor justice would have sanctioned the coercion of the individual in question, without establishing a principle that would be most dangerous and tyrannical in its nature and operation.

But to return to the history of this memorable case. In the early part of the year 1834, while in Scotland, Mr. M'Kerrell was suddenly seized with hæmoptysis, though not to any extent— and some of the most respectable medical practitioners of Edinburgh were consulted. I may mention Dr. Abercrombie, the late Mr. Turner, and Mr. Scott. The complaint subsided without any serious consequences; though there were a few very insignificant returns of it afterwards, for which I attended the patient in the succeeding year— 1835. The occurrence, however, deserves commemoration, as will be acknowledged in the sequel. As a singular feature in this case, I may observe that the patient was exceedingly alarmed and anxious, on account of a slight return of this hæmorrhage, and that at a time (the Spring of 1835) when he was meditating suicide. On carefully examining the chest with the stethoscope, and assuring the patient that there was no danger whatever, he seemed cheered up, and in good spirits. It was just about this period that he wrote to a near relation, that he was about to destroy his own life! It was at this time, also, that I strongly urged him to go up the Rhine, in company with a medical attendant, and spend three or four months among the Alps. He seemed determined to adopt this suggestion; but greatly regretted that I had not made this proposal in the Autumn of the preceding year, when he could have travelled with myself and family, in the same direction.

And now I suddenly lost sight of him, and knew not whither he had directed his steps. On the 15th of November I was summoned to him, and found him considerably altered in appearance. Although it was the "good day," he was evidently labouring under great mental depression and distress. He stated that he had been some time at Cheltenham, and had consulted an old and valued friend, who prescribed for him, but in vain, as his mental and corporeal miseries were now arrived at a degree of intensity which human patience could not long bear, nor human strength resist. He had got much thinner, and complained that some aperient medicines which he had been taking at Cheltenham, increased rather than diminished his sufferings. This, however, was probably imaginary.—From his friend at Cheltenham I learnt the following particulars.

"I had long known Mr. M'Kerrell in India, and think that much of his physical infirmity arose from utter neglect of his bowels. He would sit long in his office at Madras, or take home his public papers, and pore over them late and early, scarcely allowing himself any exercise, and what little he did take, was in solitary rides or rambles. He perspired most profusely, and constipation went to a great extent. Another of his friends and myself invited him to Cheltenham, for change of air and scene. He visited me first on the 20th of September, giving me a long history of his troubles and sufferings. He remained here till the 11th of November, having suddenly, on the 9th, taken his place for London; but repenting him of the act, changed his mind, and forfeited his fare. Again, on the 11th, he took his place in a morning coach — forfeited his fare a second time—and went up to town by the night coach. During one week of his stay here, he had a gleam of hope that his health would be re-established, and was only one day in bed that week. In the next week, his gloom came over him, and he did not leave his bed for three days. All this time, however, his desire for food was more than natural, and he never, even on his bad days, ate less than from two to *three mutton chops at breakfast, and a large meal of animal food to dinner.* I occasionally passed an hour with him, *on his bad days,* when he evinced an unnatural degree of calmness—always expressing himself as tired of life, and only restrained from suicide by the fear of God's wrath."*

It will be remarked in the above statement, that to his medical attendants (myself excepted) he would give access on the bad days. To me, who was acquainted with the illusion, he would never give an audience at such times. This is very strange.

The remainder of the tragic scene is well known to the public. When I parted from him on the 15th of November, being his "good day," though a very wretched one, he desired me to call again on the 25th of the same month, which would also be his "good day." On Tuesday, the 24th, however, being the day of the illusion, he wrote a short letter to Mrs. Vickery, his landlady, announcing his determination to commit suicide, and giving her some directions about his keys, and the place of his sepulture. He rang the bell about four o'clock, and ordered a wine-glass. At a quarter before seven o'clock, he was found lying on the floor, dead, and nearly cold—with an empty two-ounce phial labelled "hydro-

* Mr. M'Kerrell himself frequently acknowledged to me, that before his return from India, he laboured under a periodical affection, of which he could give no accurate description, except that he felt alternately (but not very regularly) ill and well on particular days. His spirits were depressed, even then, on the bad days. I learnt from a lady who came home in the same ship with him, that she distinctly recognized the good and bad days, by his conduct and appearance at table, as he always sat next her.

cyanic acid," of Scheele's strength, on the table, standing beside the empty wine-glass. In the bottle, there was a drop or two of the acid, exhaling the odour of almonds!

An inquest was held the next day, before Mr. Gell, and twenty respectable jurors. I stated as much of the foregoing narrative as I thought necessary for the ends of justice, and repeated twice or thrice over, that the suicide was committed *on the day of the illusion*, as proved by my visiting-book, which I produced in court. Having retired, after giving my evidence, the coroner, labouring under the most unaccountable misapprehension, represented to the jury that, according to my evidence, the suicide was committed on the "good day," or the day on which the deceased was free from illusion!! I do not accuse the coroner of wilful misrepresentation of my evidence—but still the *misrepresentation* was not the less real—nor the *misdirection* to the jury. Had the whole, or even a majority of the jurors, been guided or influenced by the charge of the coroner, the consequences might have been most disastrous—the forfeiture of a large fortune to the Crown—and the affixation of an indelible stigma on the moral and religious character of an unfortunate sufferer from mental and corporeal disorder! Fortunately for justice and humanity, the testimony of the physician outweighed the judicial authority of the lawyer, and fifteen out of twenty of the jurors brought in a verdict of "temporary insanity," instead of FELO-DE-SE. I stated in the public papers, at the time, this misdirection of the coroner, and he has not thought proper to impugn the statement—for the best of reasons—because he could not.

*Dissection, 27 hours after Death.*

The body was examined by Mr. Henry Johnson (my son), and Mr. Bushel, in presence of Dr. Robert Lee, Mr. Harding, and myself.

The emaciation was very great. The deceased having fallen on his face, there were marks of contusion about the left temple, and the face itself was rather of a bluish hue.* The body exhaled an odour of Prussic acid. The eye did not present any particular appearance. The stomach was remarkably capacious, and its contents were sent to Dr. Turner, for analysis. In some portions of the mucous membrane of this organ, especially near the upper and inferior orifices, there were marks of recent inflammation, particularly in stellated patches, where slight marks of extravasation were perceptible. In several portions of the small intestines the external hue was dark, almost approaching to livid—and the mucous membrane of these portions was vividly red, or of a dark red colour; but without extravasation, or perceptible injection of the vessels. It resembled imbibition, or that which would be produced by steeping sound, yet dead parts, in blood. The large intestines were greatly distended by air, but exhibited no marks of disease of any kind.

The liver, spleen, mesentery, kidneys, and all the abdominal viscera were perfectly sound.

Before a knife was laid on the body, I expressed a wish that the ganglionic centres might be carefully examined, as I conceived that some irritation of the nerves of organic life played an important part in the phenomena exhibited by the patient. The solar plexus was therefore minutely investigated; but nothing abnormal was perceptible.

In the thorax there was great and varied disease. The lungs were studded with tubercles, especially the superior lobes, and extensive adhesions existed between the pleuræ-costales and the

* I was informed by Dr. Watson, the intelligent physician of the Middlesex Hospital, that, in two cases of poisoning by Prussic acid, the face exhibited an appearance very similar to that of scarlatina. The circumstances attendant on the present case (the falling on his face, and the contusions about the head), would probably have prevented the phenomenon observed by Dr. Watson.

p. pulmonales. None of the tubercles had broken down, so as to discharge their contents through the bronchial tubes.

The heart was not larger than natural—or very little more than the ordinary size; but the pericardium was universally adherent, by old, that is to say, by cellular attachments. The organ itself presented one of the finest specimens of "simple hypertrophy," which we have ever seen. The parietes of the left ventricle were an inch and a quarter in thickness; and the cavity was, with difficulty, discovered. It could not have contained more than four drachms of blood, if so much; which is scarcely one-third of that which a normal left ventricle would be capable of throwing out at each contraction.* There was nothing abnormal in the arteries. The blood in every part of the body was perfectly fluid, and exhibited not a single coagulum in any vessel.

The brain was remarkably firm, and the vessels were rather congested; but there was no visible trace of disease in the head. The skull was of unusual thickness and density.

And now comes the most remarkable portion of the pathology. Upon the nervus vagus, or pneumo-gastric nerve of the left side, just before the recurrent is given off, there was affixed a hard jagged body, the size of a kidney-bean, or small nut, composed of calcareous matter, and probably a diseased bronchial gland converted into this substance. The union of the nerve and this rugged mass was so intimate, that no dissection, without cutting the nerve or the calcareous body itself, could separate them. The foreign body, in fact, had penetrated, or at least invaded the nerve, which was thickened at this part. Lower down, and involving the cardiac, pulmonic, and œsophageal plexuses in a labyrinth of perplexity, were several diseased bronchial glands, rendering the dissection a tedious and difficult operation. The parts were all carefully removed, and the investigation conducted slowly, and at different periods, in the Kinnerton-street Theatre of Anatomy, by Mr. Johnson, Mr. Tatum, Mr. C. Johnson, and others. The preparation is preserved in the museum, and may be seen by any gentleman there.

When we consider that the nervus vagus rises in the medulla oblongata, but is chiefly distributed to the great organs *not* under our control—and that it communicates with almost the whole of the ganglionic nerves, we may form some idea of the irritation and disturbance produced in the digestive, sanguiferous, and sanguific organs, by a jagged calcareous mass implanted, as it were, into one of the most important nerves of the great vital viscera! If this pathological phenomenon be held as nought in the production of functional disorder, then I say that we had better lay aside morbid anatomy entirely, and go back to the old Hippocratic practice of watching the symptoms—employing few or no remedies—and recording the death or recovery.

I am well aware that here a very feasible objection may be made to the operation of the pneumogastric irritant, as connected with the phenomena observed during life. The irritating cause was permanent—the effects (if they were effects), were periodical. This objection is, I confess, somewhat startling, at first sight. But let us examine it a little more closely. Do all physical agents produce permanent, or, at least continuous effects?—A man residing in the marshes of Walcheren or other malarious locality, becomes affected with ague. He is every day exposed to new malaria, and yet he is only *every other* day influenced by it.—How is this to be explained? But in the whole class of nervous affections, we find, in practice, a strong disposition to *periodicity*, though the *cause*, as far as we know, is *constantly* applied. Thus tic-douloureux, tooth-ache—and the extensive tribe of neuralgic pains, are all, more or less prone to shew themselves periodically, although we have not the slightest proof that their causes are only periodically in operation. We

---

* A section of the ventricle was seen in the Kinnerton Street School of Anatomy by many medical gentlemen.

know that visceral diseases will keep up affections of a periodical type, long after the individual is withdrawn from the sphere of the original cause. This was witnessed on a large scale, ever since the fatal expedition to Walcheren: and it is familiar to all those who have practised in malarious countries.

In the present melancholy case, there was ample visceral disease to produce hypochondriasis, monomania, or insanity, in an individual predisposed to such affections. It is curious that the majority of the organs to which the pneumogastric nerve distributes its influence, were changed in structure, or disordered in function. The state of the heart must have had a great share in deranging the general health, and probably accounts for the excessive emaciation, although large quantities of animal food were daily consumed. The heart could not circulate more than one third the usual quantity of blood through the lungs and the body generally. This must have produced a deficient sanguification in the pulmonary apparatus, whatever was the amount of digestion and chylification. The functions of the stomach were remarkably deranged. Indeed this unhappy man laboured under a kind of bulimia. Diseases of the heart are very apt to affect the mental functions through the instrumentality of the brain; and upon a careful consideration of the whole case, I think I am authorized to conclude that, the mental functions, as manifested by their material organ, the brain, were disturbed by the physical changes found on dissection, and that the monomania in this instance, as probably in most others, was dependent on corporeal rather than on moral causes.

The publicity given to this case by the inquest has led to several communications disclosing other cases more or less similar, three of which are now living. The names and particulars of these cannot here be stated; but one communication deserves particular notice. The communicant is a professional man, and of great experience. He moreover affirms that he has suffered for many years, in his own person, very nearly in the same way as Mr. M'Kerrell. He has also become acquainted with several other instances of a similar kind. He affirms that, in every case, including his own, there was some consciousness, or at least conviction of GUILT. He does not believe that the *guilt* was always a reality—or that when it did exist, it was of a kind that need have produced such repentance or remorse; but still he maintains that, from a professional (I do not say whether it was medical or clerical), acquaintance "with a vast variety of strange cases of mental dejection, despondency, and misery, there was, in every instance, something, more or less, of moral guilt." He relates the case of a lady who, for forty years before her death, laboured under a state of "black despair," yet never revealed the *cause*—although there was a cause—and that connected with a feeling of moral guilt.

There may be some truth in these observations of my veteran correspondent; but my own experience convinces me that for every one instance of remorse or repentance for *real guilt*, leading to monomania, there are fifty *imaginary* ones. In the present case I can solemnly declare that the illusion had nothing to do either with real or imaginary crime.

Early in the present year, a most remarkable case of this nature came under my observation. A gentleman of considerable talent and ingenuity, who had overworked both mind and body in an avocation imposed on him by Government, became affected with a periodical complaint of a most distressing nature. He was brought to me by his own physician residing in the city. The disorder had continued for more than two years. On one day, he had great energy and excitement of mind. Every thing was *couleur de rose*—his affairs were most prosperous—and his family most amiable and obedient. On that night he got very little sleep, the excitement of mind preventing repose. After a short and troubled slumber towards the morning, he awoke to a day of the greatest misery. The whole prospect of the preceding day was reversed.—Black melancholy or perturbed irritation of mind occupied him. His affairs were all going to ruin, and his family must, inevitably, come to the workhouse—his previous speculations were all on the wrong side—and his family were precipitating his downfal by misconduct!!—In this state he was persuaded to make a tour up the Rhine and through Switzerland. The contrast between his good and bad days gradually lessened, and while he was using strong exercise among the high Alps, he could scarcely distinguish the one day from the other. On his return to London, the periodical miseries also recurred; and, on examination of his notes, he found that the periodicity never altered his regularity of type—namely, that the bad day was precisely the same as if the chain had never been broken by the excursion.

Although the sombre day presented a number of triste emotions and melancholy ideas, there was ONE predominant misery which formed the burthen of the song. It was of a domestic nature, and need not here be stated. The case was clearly monomania, and I was consulted on the medico-legal point in particular. I was of opinion that the gentleman could not be deprived of his liberty; but fortunately he agreed to give up the management of his affairs to proper persons, and thus prevented the ruin of his family and the publicity of the evil.

Though not at liberty to divulge the exact nature of the illusion, I may fairly illustrate the mode of its operation by *fictitious* ones. Suppose an individual afflicted with bodily disease, became affected, every second day, with the following illusion. The moment the sun appeared above the horizon, a horrible face presented itself in the disk of the luminary, frowning upon the individual, and menacing him with destruction—this spectre being also attended with the most horrible feelings of body and mind, which no language could describe. This was *not* the illusion of my patient; but this was the way in which it acted. Suppose, again, that there was a certain colour—say scarlet—the sight of which caused the most dreadful anguish and despair, on alternate days, although the same colour could be viewed, without emotion, on the intermediate days. This was *not* Mr. M'Kerrell's illusion, but this was the mode of its operation. Suppose the figure 9, or any of its combinations, when presented to the eye, caused the miseries so often mentioned, and that only on alternate days. This was *not* the number; but the effects were the same. Now what possible connexion can any of these have with real or even imaginary guilt?

I am informed by a highly respectable medical gentleman in Edinburgh that, at the moment I am drawing up this paper, there is a gentleman at Leamington affected precisely in the same manner as Mr. M'Kerrell was affected. With the illusion itself I am unacquainted. The same communicant informs me that a late eminent veteran professor of medicine, whose name I am not at liberty to mention, laboured under a similar malady for several years before his death.

The chief peculiarity of the case narrated, is its PERIODICITY. But when we consider that, in a great many cases of insanity, there are *lucid intervals*, though not quite regular—and again, that a vast number of bodily diseases are periodical, we have no reason to doubt the truth of the instance which I have detailed, authenticated as it is, by so many different individuals, both in and out of the profession.

Nor is it entirely without a parallel, as to periodicity, in recorded annals. Pinel informs us that, out of 200 maniacs in the BICETRE, there were 52, whose paroxysms recurred and intermitted at irregular intervals—and six in whom the periods of accession occurred *regularly*. Among the *latter* class was one, whose paroxysm returned every year, and lasted for three months, ending regularly towards the middle of Summer. A second was subject to extreme fury during a fortnight every year; being perfectly calm and rational for the remainder of the time. In a third, the disease observed the type of a tertian fever, *there being one day of complete intermission.**

M. Foville mentions the case of a young woman who was periodically insane—each paroxysm and each interval being 14 days.† These instances are not less remarkable than the one which forms the subject of the present paper.

The following case was communicated to me, orally, by my esteemed friend Dr. A. T. Thomson. A couple were to be married, and the nuptial day was fixed. On his way to the bride's house, the bridegroom fell from his horse, and was killed on the spot! The fatal intelligence caused a severe and dangerous illness in the widowed maid. On recovering from this, she became affected with a very singular species of periodical monomania.—Every day, and about the hour of the day when she first received the dreadful intelligence, she fancied that she saw her intended spouse descend from the skies. She was then in a species of extacy, with her eyes rivetted on the Heavens. Presently he appeared to catch her in his arms, and ascend into the air. She was then in a state of violent agitation, amounting almost to convulsions. When at a certain height, she fancied that her earthly frame became too heavy for the spirit that was bearing her away from this globe, and she fell from her lover's grasp. She was then in a kind of swoon, in which she continued for an hour or two, with scarcely a sign of life. The whole paroxysm lasted for some hours, when she became restored to reason. The monomania returned every day at the same hour—and the sequel was not known.

A somewhat similar case was the following. A man who was absent from home, for some time, wrote to his wife that he would return by a certain coach, and at a certain hour. She went to the coach-office to meet her husband. She there learnt the astounding intelligence that he was killed on the road. She fell into a severe illness, on recovering from which, she became affected with monomania.—She went every day, at the same hour, to the coach-office, and after waiting for some time, concluded that her husband was accidentally delayed, and returned home—when the delusion vanished, and she became conscious of her widowhood!

* On Insanity, p. 13.

† Dict. de Med. Tom. 1. p. 525.

---

EFFECTS OF SOLITARY CONFINEMENT ON THE HEALTH OF SOLDIERS IN HOT CLIMATES. By Assistant Surgeon MALCOLMSON, late Secretary to the Madras Medical Board.

[*Letter to Sir H. Hardinge.*]

The author sets out with the confession that he is no advocate for *corporal* punishment, being satisfied that it is not effective in the prevention of military crime. Yet he cannot shut his eyes to the fact, that the anxiety to avoid the revolting spectacle of flogging is likely to lead to another kind of danger—the substitution of a solitary confinement that is incomparably more cruel at the time, and more detrimental to health afterwards. Mr. M. has reason to believe that "more real misery has arisen in 12 months from imprisonment in the jails of India, than has been inflicted by corporal punishment in a hundred years." Here our author adduces some statistical reports shewing that the mortality in prisons varied from 24 to 57 per cent. per annum! Punishment he acknowledges to be necessary, flogging disgraceful, and solitary confinement destructive of health; but he is unable to suggest a substitute for either the one or the other mode. Mr. Brown has a doctrine which, if taken in an unrestricted sense, is rather startling. "There can be no doubt of the truth of the principle, that *no punishment* can be just, or in the eye of God, lawful, which tends to impair the efficiency, injure the health, and shorten the life of the soldier—or which produces any effects that cannot be estimated by the judges when they assign a punishment for an offence." A soldier mutinies, and he demurs to any punishment that may injure his health or shorten his life! Greenacre and Meunier might have urged this doctrine against their sentence, as tending to injure health and shorten life! Johnson defines "punishment" as the infliction of pain or death, as atonement for crime. No infliction of this kind, to be of the least efficient amount, can be otherwise than injurious to health, in a greater or less degree. Nor can the judge always predicate or calculate the effects of corporal punishment.—What may be borne with impunity by one man, may be the death of another. Daily experience shews that the judge or the court-martial cannot estimate the amount of punishment which a man can bear, otherwise a surgeon would not be ordered to attend the infliction of the sentence, and arrest the whip when he finds the culprit sinking under its effects.

By these observations we do not mean to advocate solitary confinement and bread and water, as punishments for soldiers in the East Indies, as the injury may be more than was designed, and consequently more than is proper. But we maintain that all punishments are more or less injurious to physical health, and that flogging is a punishment much more detrimental to the moral man than solitary confinement. In the former case, the man feels degraded, and too often loses all desire to retrieve his character. In the latter, he is not exhibited in public, and has ample time for reflection and repentance in his lonely cell, where his messmates cannot pamper him with intoxicating draughts. But while we advocate seclusion, we condemn starvation on bread and water. We would mortify the spirit, but not withdraw proper sustenance from the body. If the great object of punishment be the prevention of crime, we know, from observation, that the dark cell is more dreaded than the whip. This we saw exemplified among the French prisoners during the last war. Nothing was so operative in preventing crime as the dread of the CACHOT.

Our author's attention was first drawn to the effects of this kind of punishment by two men who were so treated at Hyderabad. These effects resembled a good deal those of sea scurvy. The history of the Penitentiary Endemic, by Dr. Latham, also exemplifies these effects.

"Many men, particularly those of indolent habits, endure a confinement of four or six weeks, on bread and water, without injury to their health; but, in some instances, a shorter period is sufficient to cause a total loss of appetite,—the bread is hardly touched, and on other food being allowed, the patient is unable to eat or to digest it. The stomach becomes weak; there is uneasiness across the region of the stomach, spleen, and liver; the latter is torpid; the bowels are confined, or they are relaxed with slimy discharges unaccompanied with pain, yet the swollen red tongue indicates the existence of irritation of the mucous membrane of the digestive canal. The pulse is quick and feeble; and the clammy skin, vertigo, debility, headach, and sleeplessness, show how much the constitution suffers from diminished nervous power. The convalescence is slow, and the treatment requires to be adapted to the enfeebled state of the system. The effect is, however, more clearly seen in men sentenced to six or twelve months solitary confinement. Two of these were in hospital at the same time, with decided symptoms of scurvy:—one was admitted after five months confinement, during part of which he had been allowed extra diet at my recommendation. It was observed, that for some time previous to his removal to hospital, his daily allowance of bread was removed almost untouched. He complained of pains of the limbs, along the spine, and across the loins; tenderness of the shin bones; hardness, pain and feeling of stiffness of the calves of the legs, and the skin over the painful muscles was of a dark livid colour from effused blood. The gums were spongy, livid, and retracted, and he suffered from sleeplessness, some pain of the region of the liver, and slight griping. The tongue was yellow and its edges red. The other had been a shorter time in confinement, and complained of debility, disorder of the bowels, pains of the shin bones, &c."

We wonder indeed how any rational people could think of sentencing soldiers to six or twelve months' confinement on bread and water! The plan must prove ruinous to the constitutions of nine-tenths of the miserable victims of a mistaken and too rigorous system of retaliation for crimes not capital.

---

### NITRATE OF SILVER IN IMMENSE DOSES.

There resides in the vicinity of London a gentleman whose face must be familiar to many thousands of its inhabitants. His skin is intensely blue from the internal use (or abuse) of nitrate of silver. He is now about 73 years of age, and at the age of 45 became affected with epileptic fits of the most violent and distressing kind. In these attacks he struggled so strongly that it required two or three people to hold him. The fits also were very frequent, and in one of them he totally and for ever lost the sense of hearing. A most distressing noise in the head, however, has even since harassed the patient. After trying various remedies for the epilepsy, without the least benefit, he was advised by the late Dr. Curry, of Guy's Hospital, to enter on a course of the nitrate of silver, beginning with small doses, a quarter of a grain three times a day, gradually increased. In the course of a few weeks the attacks began to diminish in intensity, and in a few months, the intervals became much lengthened. The medicine was continued in augmenting doses till the epileptic paroxysms entirely ceased, which they did within six months from the commencement of the course of medicine already mentioned. Dr. Curry never warned the patient as to the effect of nitrate of silver on the skin, if long continued, and the physician himself dying about this time, the patient persevered with the medicine for the space of three whole years, the dose being eighteen grains a day for nearly all the third year! The patient, who is a highly intelligent man, avers that he never experienced any unpleasant effect from the medicine—indeed he felt no effect at all, except its remedial power in arresting the epilepsy. Not having any idea of its physiological agency on the skin, he continued the nitrate with the view of effectually guarding against the return of the malady which he so much dreaded. It does not appear that the blue colour made any serious advance till towards the close of the third year, otherwise it is probable that he would have taken alarm, or that he would have been apprized of the circumstance by his friends. At this remote period, however, he cannot distinctly recollect the exact time when the blue tinge of the rete mucosum commenced or acquired its complete acmé of intensity. This fact is of considerable interest, as there is probably no case on record where such a quantity of the nitrate of silver has been taken, or continued so long. It accords with our own experience as to the absence of any sensible physiological effect on the stomach and bowels which usually attends the administration of this medicine—unless we except a certain soothing agency — probably the consequence of obtunded sensibility in the gastro-intestinal nerves and tissues. In all cases excepting epilepsy, however, it is never necessary to give more than a grain or two of the nitrate daily (pills made up with extract of liquorice) and not continued beyond a few weeks at one time. Combined with the compound extract of colocynth, also, it forms an excellent remedy for irritability of the primæ viæ attended, as is often the case, with torpor of the bowels and vitiated secretions.

---

### ON THE EFFECTS OF COLD. By SIR HENRY HALFORD.

This little dissertation was one of those papers with which the worthy

Baronet has annually opened the College over which he presides, at the commencement of the London season. Few of Sir Henry's socii have been able to adapt their discourses so admirably to the mixt audience of a commencing session as the President himself. On these occasions the senator, the divine, the philosopher, and the poet are all sure to find in Sir Henry's addresses, something to excite their attention, call forth reflection, or gratify the intellectual palate.

The present Essay, though very concise, embraces many striking incidents connected with wide-wasting war or perilous adventure, from the expedition of Banks and Solander over the inhospitable tracks of Terra del Fuego, down to the disastrous flight of Napoleon from the smouldering towers of Moscow. Dr. Solander, who probably had read the Cyropædia of Xenophon, or, at all events, was practically acquainted with the effects of extreme cold amongst his native mountains of Sweden, warned his companions against the fatal lethargy that is apt to assail the snow-surrounded traveller, yet was the first to experience it, and the most urgent in his entreaties to be left to his fate!

Xenophon's retreat is next adduced by Sir Henry, and his loss of 30 men in one night, burnt by the snow storm!

" Borea penetrabile fugus aduret."

The wretched fate of the army under Sweden's mad monarch forms a fine illustration of the effects of cold on large masses of men; but the most memorable and fatal catastrophe of this kind is the one which occurred in our own times, and hurled the diadem off Napoleon's head.

From the historical part of the subject Sir Henry goes on to the pathological, and coincides with Portal of old, and Dr. Kelly of late, that cold induces death by apoplexy. The only original part of this paper is a manuscript account of the death of an English clergyman by cold in the ascent to the Col de Benhomme in Switzerland. Though accompanied by a guide and some fellow-travellers, he was obliged to be abandoned in the snow, the survivors having, with difficulty, reached the hospice on the summit of the pass! Sir Henry was informed by the late Russian Ambassador that there is a level country, about 100 leagues square, sloping to the South, on the borders of Siberia, where a year rarely passes without people dying at the age of 130! The registers are said to be very accurately kept.

Our author concludes by observing that the records of Greenwich and Chelsea Hospitals prove that the soldier lives longer than the sailor, verifying the remark of Homer three thousand years ago—that

" Man decays when man contends with storms."

There is probably much truth in the solution which Sir Henry attempts of this disparity of years in the two services. The soldier does not enter on military duty till his frame has been completely developed. The sailor hangs over the boiling surge, " on the high and giddy mast," when only a boy. No wonder that his range of life should be curtailed, when we consider the hardships which he endures, and his imprudent thoughtless conduct while on shore.

We repeat that this little oration (if we may apply the term) was well adapted to the audience before which it was delivered, though containing nothing of an original character. The historical facts were well selected, and the classical allusions, as might be expected, were elegant and appropriate.

---

### Retroversio Uteri.

In the month of March of the present year (1837) Dr. Johnson was summoned to a lady, aged about 25 years, married, and who had borne one child three years previously. She had menstruated regularly, from the death of her child, up to within a few days of the present time. She was seized, while at dinner, with severe pain in the lower part of the abdomen, between the umbilicus and the pubes, for which a practitioner prescribed some opiates. Next day Dr. J. saw the patient, and found that the pain had returned, after a temporary mitigation. On examination of the abdomen, there was some slight tenderness, but no tension, swelling, or symptom of inflammation, local or general. A brisk aperient was prescribed, and on the succeeding day there was very little pain complained of by the patient. The aperients were continued for a day or two, when the lady considered herself well, and Dr. J. discontinued his visits.

In a few days afterwards, he was again summoned, and the lady complained that she was unable to make water, except with the greatest difficulty, and in small quantities at a time. On inquiry, it was now found that this complaint had been gradually increasing since the commencement of the attack already mentioned, but she had not disclosed it till it arrived at its present height of distress. External examination threw no light on the nature of the complaint, there being no swelling, tenderness on pressure, or pain. Dr. J. attempted to introduce a female catheter, but without success. It was met by an insuperable obstruction, apparently about the neck of the bladder. On examination by the finger, the vagina was found to be occupied almost entirely by a tumour situated between it and the rectum, and rendering it difficult to introduce two fingers. The os uteri could be felt tilted up against the pubes, or rather above it, and the body of the uterus could not be distinguished from the tumour obstructing the vagina. The same tumour could be felt obstructing the rectum. An elastic catheter was, after much difficulty, introduced into the bladder, but very little water could be drawn off, as the bladder was evidently displaced. One of the most eminent physician-accoucheurs in London was called in, and after a careful examination, pronounced it to be *retroversio uteri*, and believed that the womb was in an impregnated state. All efforts to replace it were ineffectual, and the lady continued to suffer much from incessant attempts to make water. Two or three days afterwards, another distinguished accoucheur was consulted and confirmed the opinion of the former, except that he had some doubts respecting impregnation, seeing that the mouth of the womb was patulous, and no symptom of pregnancy existed. He pronounced the uterus to be enlarged, and the bladder to be displaced, as he could not introduce a catheter. He could suggest no means of relief. A few days farther on, and the first physician-accoucheur again made a careful examination, per vaginam et rectum. He was now fully convinced that at least three months' pregnancy existed. On the day that this last examination was made, the lady suddenly passed a large quantity of water, with perfect ease, and sent for Dr. Johnson. On examination, the tumour was gone —the os uteri and the uterus itself could be distinctly felt in their natural situation, and no inconvenience was afterwards experienced by the lady, who has continued to menstruate, and is in perfect health.

This is a curious and instructive case. That the uterus was retroverted, and, at the same time, unimpregnated, there can be no reasonable doubt. But that it was retroverted by the state of the bladder, and that the bladder itself formed a principal part of the tumour, *inter vaginam et rectum*, appears to the reporter extremely probable.—J. J.

---

### DR. LEWINS ON COLCHICUM.

A paper on this subject appears in the last Number of our Northern contemporary. Dr. L. appears to think that—" a great proportion of the medical profession in Great Britain are ignorant of the medicinal virtue of colchicum, or at least underrate its efficacy, whilst many decry it as a drug of doubtful and dangerous character." We know not how this may be North of the Tweed—but, in the South, we can assure Dr. L. that there are few medicines in the Pharmacopœia, with the exception of calomel, senna, salts, and half-a-dozen common drugs, which are more frequently seen in prescriptions than colchicum.

Observing the effects of this medicine in gout, and its power of diminishing irritability of internal organs, Dr. L. thought it might be useful in all or many inflammatory diseases—a conclusion to which most practitioners in this part of the kingdom have long come. And here we may correct a singular mistake into which our author has fallen. He remarks that the London and Dublin *Pharmacopœias* direct, the former two drachms, and the latter four drachms of wine of colchicum for a dose. Now the London Pharmacopœia never mentions the dose of any medicine—and we believe the same of the Dublin. In order to ascertain the effects of this medicine, our author instituted a series of experiments on himself and others in health—imitating the practice of a countryman of his own, who used, some thirty years ago, to give digitalis parties to students.

The first experiments which our author appears to have made, were on himself. He took from forty to seventy-five *drops*, without feeling much other sensation than that of depression. To a medical student he gave, in twenty-four hours, 160 *drops*, with the effect of slight nausea, followed by sleep. Six or eight watery motions, however, with considerable griping, and "great commotion" in the colon, were among the physical effects. These are just the common phenomena which we observe when the dose is a large one—namely, a fluid drachm, which contains at least 100 drops.

After several other experiments, our author began to exhibit colchicum in fever. He first ordered a female servant to take thirty drops every three hours; but rather violent vomiting came on, and the dose was diminished one-half, and ultimately to ten drops every three hours. The effects were salutary—sweating, vomiting, purging, &c.—with reduction of the fever. Six cases are detailed, of which the one here noticed may serve as a sample.

Dr. L. intends to renew the subject in a subsequent Number of our contemporary; meantime he comes to the conclusion that, from the results of his trials hitherto, "we may more certainly cut short fever, or at least break its force, by the judicious administration of colchicum, than by any other known means." To this we respectfully demur, and would submit that, by employing colchicum as an *auxiliary* to other means commonly in use, we will succeed better. Dr. L. indeed, does not prohibit other means, but he thinks that "they will seldom be required if colchicum be boldly but prudently prescribed in the early stages of disease." Dr. L. in another place, tells us that every dose must be carefully watched by the practitioner, and increased or diminished according to its effects. But we ask Dr. L. what sort of practice he is in, when he can afford to dedicate almost the whole of his time to a single patient? If an epidemic fever, or any other febrile complaint prevailed, Dr. L. would require at least a score of assistants to watch his colchicum patients! People should have reason in their recommendations, and remember that every medical man cannot sacrifice himself and his family, by devoting the whole of his time to the attendance on one or two patients. But one medicine may be very usefully employed in a great many febrile and inflammatory complaints, without all this precaution, and yet with very sensible and unequivocal effects. The dose, in general, may vary from ten to thirty minims, though the latter may be considered a large one, except when given for gout or rheumatism, and that at bed-time, without any other dose in the day. When given for common inflammatory affections, ten minims three times a day to a grown person, will be found quite enough; and we imagine that it is much better to give this active medicine in *such* doses, and in combination with other remedies of known efficacy, as antimony, digitalis, calomel, &c. than to exhibit large and powerful doses of colchicum *alone*, requiring constant attention to prevent injurious effects.

But there is one other consideration worthy of attention. When the abnormal or poisonous effects of colchicum *happen* to take place, have we any certain means of controlling them? We apprehend that we have not such means. Opium and cordials may sometimes check the enormous vomitings and purgings of colchicum; but we should be very sorry to incur the risk, while trusting to these correctives. Dr. L. mentions a case where two drachms of the medicine proved fatal lately in a public hospital.

---

### Malignant Disease of the Stomach, attended with remarkable Symptoms.

Mr. J———, about fifty years of age, and reported to have been active and temperate, came under Mr. Diamond's care about four years ago. His chief complaints then were diarrhœa, and discharges occasionally of blood from the bowels, of a florid colour, supposed to be hæmorrhoidal. On the 24th of July, 1836, Mr. J. was suddenly attacked with a most violent pain in the chest, attended with sense of suffocation, and a feeling as if he was dying. These attacks recurred frequently, and the advice of an eminent surgeon was taken. This gentleman considered that the disease was angina pectoris. The patient, at this time, complained of no pain in the stomach or sickness, nor did pressure in that region occasion any inconvenience. He appeared to improve in health, and went to Margate. On the 20th of October last, Mr. Diamond was summoned to the patient, and found him labouring under an attack of the complaint believed to be angina pectoris. No medicines appeared to give any permanent relief. He now began to emaciate much. About Christmas he complained of a burning heat in his stomach, for which Dr. Elliotson prescribed twenty-five grains of the subnitrate of bismuth three times a day. He seemed worse after this. On the 23rd of January, 1837, Dr. Johnson saw the patient, and on examination, discovered a tumour in the region of the stomach, which he considered as of a malignant nature, and that it would be fatal. He prescribed, as a placebo, a mixture of lac. amygdal.—carb. magnesiæ, mucilage, and tincture of hyoscyamus. This appeared to give him much relief as far as the stomach was concerned. There was no vomiting. But the most formidable symptom was now a periodical attack of what resembled violent chorea, which came on regularly every seventh day, namely, on Sundays—and sometimes required one or two people to hold him in bed. These fits recurred for six weeks before his death. Its last accession was out of its usual course. It occurred on a Thursday, and on the following Sunday he died. It was evident, on dissection, that the fatal event was occasioned by internal hæmorrhage; for the intestines were found to be full of coagulated blood, and all the vessels of the body were completely empty. The periodical attacks of violent jactitation did not seem to be occasioned by food; for in one of the severest that Mr. Diamond witnessed, no food had been taken for six hours previously to the seizure.

*Concise Account of the Stomach.*

The *lesser omentum* was quite obliterated, and the left lobe of the liver intimately adherent to the *smaller curvature* of the *stomach.*

The *greater omentum* was much thickened in the neighbourhood of the *greater curvature* of the *stomach,* and some of the thickened portion had turned over the anterior surface of the *stomach,* and contracted an adhesion to the *smaller curvature.* The consequence was, that the *stomach* formed a sort of pouch on the right, and another pouch on the left, while the adherent band of omentum produced an hour-glass contraction in the middle.

On opening the *stomach,* a small portion of its anterior, and a large portion of its posterior surface, nearly the whole of its *lesser curvature,* and the vicinity of the *æsophageal opening* were the seat of *fungoid tubercles* more or less ulcerated, and partially sloughing. In other parts, the tubera were in an earlier stage. In these spots the muscular coat of the stomach was hypertrophied, and undergoing the malignant change; while between the muscular and mucous coats, the fungoid deposit had commenced. A section of one in a more advanced condition, exhibited incipient softening in the centre, and ulceration beginning to invade the mucous membrane.

The character of the morbid growth was neither that of pure scirrhus nor of pure medullary sarcoma—but, as not unfrequently happens, of an intermediate production. Such is occasionally seen in the breast, the liver, and the stomach—and, indeed, in the other organs and tissues of the body.

It is not very easy to account for the periodical attacks to which this unfortunate gentleman was liable. Still, when we contemplate the amount and the nature of the organic disease in so vital an organ as the stomach, the wonder is that he lived so long, or that any digestion at all could be effected. The absence of vomiting in this and in other organic diseases of the stomach, where the pyloric orifice is free, and the cardiac involved in disorganization, is not inexplicable.

---

### Sir Francis Smith on Creosote as an External Application.

The observations of Sir Francis Smith were originally read to the College of Physicians in Ireland, and have been published in our esteemed contemporary of Dublin.*

Sir Francis observes that practitioners in this country evince an indisposition for the use of new remedies—an indisposition, which, he thinks, amounts to a vice, although in its origin a virtue. We feel some doubts of the correctness of Sir Francis's opinion on this point. We see, in practice, in London, little evidence of the bashfulness which he deplores, nor of that coy reluctance to prescribe new drugs, which in Ireland would seem to amount to a vice; nay, could we trust the evidence of our senses, we should rather deplore the wholesale manner in which the new medicines are abused, and the want of discrimination, we might sometimes say of common sense, which is evinced in their selection or their distribution.

Sir Francis has not made many experiments on creosote, and those experiments have been confined to its external application. He observes that one source of discrepancy in results will exist in the difference of specimens made use of; the strength or concentration differing much both from the variety in the mode of preparation and purification, and also from the mode of preservation. That which he has always used was from the Apothecaries' Hall, of sp. gr. of 1,064, and nearly colourless. He has tried the creosote in three cases, or rather, he relates three cases in which he has tried it.

*Case* 1. A gentleman had phymosis, with the outside of the prepuce covered with ulcers of a ragged phagedænic appearance, with a good deal of constitutional disturbance. Some alterative doses of blue pill, in combination with morphia and tartar emetic, were prescribed, with soothing applications to the parts. This treatment had the effect, in conjunction with the recumbent posture, of quieting the constitution, and diminishing the tension of the parts. But every mean was tried in vain to cause the ulcers to heal; they still preserved their phagedænic character, and at length united into a zone, nearly surrounding the prepuce, and threatening its destruction. Sir F. Smith now applied the creosote pure with a pencil. Next day the sores were improved, and had contracted one-third in diameter. Being lightly touched every day for six days, they were entirely healed.

*Case* 2. A young gentleman, much broken down in constitution, became affected with an abscess by the side of the anus, which opened near the latter. On passing a probe into the orifice, says our author, "I found an orifice through which the matter, which amounted to an egg-cup full, had been evacuated;

* May, 1837.

and on passing in a probe, I found it to enter about one inch, passing upwards, and resting upon the side of the gut; on further investigation, I found that the sinus branched about midway its course into another cul de sac, which passed rather away from the gut, and presented to the probe a rough, yielding, spongy sensation; at the opposite verge of the anus an inflamed pile existed." The soothing plan was continued, but it did not answer. Under these circumstances, the creosote was resorted to. Sir F. introduced to the bottom of each cul de sac a very small dossil of lint smeared with it. In two days more the creosote was repeated, and four more applications spread over a fortnight, nearly perfected the filling up the sinus. The red precipitate afterwards completed the cure.

*Case* 3. A lady, of a scrofulous habit, had had for some months ulcers of the septum narium, which had resisted much and varied treatment. Sir F. resolved to do nothing but employ the creosote locally.

"The ulcers amounted to four, varying in size from the head of a pin to the section of a very large pea; three on one side of the septum, and one (the largest, and the first which had made its appearance), on the other side.

I at first made use of a wash, consisting of one part creosote, with sixty of water; in which proportions they unite at common temperatures; the wash to be snuffed up the nose frequently in the day. The odour was complained of as very disagreeable, and at the end of two days, I was disappointed by finding that I had made no progress in improving the appearance of the ulcers. I now determined to apply the creosote in its pure form, and began by pencilling the edges of the ulcers with a brush smeared with creosote, and directing the patient to inhale the fumes of acetic acid for a few seconds subsequently. The application of the pencil was rendered easy by firmly grasping the ala nasi, and drawing it outwards; and I advised the inhalation of the fumes of acetic acid for two reasons: first, because acetic acid is the proper solvent of creosote, and would, by being inhaled immediately after its application, have the effect of rendering its action more equable and uniform; and secondly, because the odour would tend to counteract the disagreeable fuliginous flavour of the creosote. The next day I had the gratification to find the character of the ulcers improved; the edges were much less abrupt, and I now determined to apply the creosote lightly over the whole of the ulcer on the left side of the septum, and to brush those on the right side with a solution of creosote in twenty parts of acetic acid; and I continued to do so on alternate days for a week, at the end of which time, the ulcer on the left side of the septum was reduced to a mere point, having every appearance of immediately healing; whilst those on the right side, though improved in appearance, having smooth edges gently declining towards the centre, still preserved their original dimensions. I now applied to them also the pure creosote, repeating the application on alternate days, accompanied with the inhalation of the fumes of acetic acid. The rapidity with which the ulcers now healed was truly wonderful. That on the left side, to which I first applied the creosote in a pure state, was completely healed in ten days; and those on the right side, in six days after the first application of pure creosote, and sixteen from commencement of treatment."

Sir Francis observes that for small ulcerations in the mucous membrane the pure creosote is most effective—that the solution of one part in twenty of acetic acid is not destitute of power—but that the solution of one part in sixty of water is only adapted for ulcers where the surface is large.

There can, we think, be little question that the creosote is an useful application where a powerful stimulant is wanted. It must be classed as a local agent with the balsams, the compound tincture of benzoin, &c. Probably those surgeons who possess the greatest amount of experience will be least disposed to attach to it any very marvellous properties.

### Dr. Lendrick on the Use of the Nitro-Muriatic Acid Bath.*

Dr. Lendrick is of an opposite style of thinking to Sir Francis Smith. He is not afraid of an excessive aversion on the part of the profession to new remedies. "I have often," says he, "inculcated on my pupils, the importance of accurately scrutinizing the merits of obsolete modes of treatment, rather than speculating on new and untried remedies; under the conviction, that there is little recommended on respectable authority, which is not of efficacy in *some* cases."

Accordingly Dr. L. writes strongly in favour of the nitro-muriatic acid bath. He thinks the neglect into which it has fallen arises in a great degree from its having been used only in the form of a foot-bath. He advises its being more efficiently employed as a general bath, and he gives these directions for its use.

"The patient is placed in a common warm bath, at a temperature of from 90° to 95° (according to his feelings), twice or thrice a week, and for a period of fifteen to twenty minutes. Into each bath (of from thirty to forty gallons of water) are poured from an ounce and a half to two ounces of concentrated nitric, and from two to three ounces of muriatic acid; the proportion being nearly that of two to three.

This practice may be continued for weeks or months. The use of the bath does not seem to produce either debility or any other derangement of the general health; nor have I seen ptyalism or those eruptions of the skin described by authors as its effects, except once, and that was when it was used in a less diluted form as a foot-bath. Idiosyncracies will, however, occasionally produce anomalous symptoms, as in the case of other remedies. Its application ought, therefore, always to be superintended by a medical practitioner."

Dr. Lendrick particularly recommends this bath in what is called "liver consumption," and in the cachectic cases, which result from the abuse of mercury, or depend on the combined action of syphilis, mercury, and constitutional idio-syncrasy.

* Ibid.

---

### Dr. Fingerhuth on Hypertrophy of the Mammary Gland.

For the translation of this memoir we are indebted to our able contemporary, the Dublin Journal, in the number of which for May, it is contained. We shall not, nor need we, do more than present an abbreviated account of the memoir, which itself is rather lengthy.

Dr. Fingerhuth distinguishes two forms of Mammary Hypertrophy:—one which runs its course quicker, and is always a disease complicated with and occurring synchronous with puberty; and another which developes itself more slowly, advancing by hardly perceptible growths, and which is principally affected by disturbance in the functions of the generative organs.—The Doctor, however, confines himself to the description of the first form.—The following is his account of its course and appearances.

"The mammary gland enlarges, usually the right, rarely both at the same time, preceded by a prickling sensation, accompanied with irritability in the affected breast. The swelling extends uniformly over the entire breast. This ailment always occurs at the period of puberty, and with the development of the breast. Usually the persons affected have not menstruated, or if the menses have appeared, they flow sparingly, are of short duration, and soon cease, never to re-appear. We remark at this period a quicker growth of the swelling, at the very time when the monthly period has ceased, and very often the patients complain of a sensation of the increased tension, which, however, becomes less at the cessation of this period, and the accelerated growth of the hypertrophying organ returns to a more slow and steady progress. Sometimes now, also the voice, undergoes a peculiar change; it becomes rough, hoarser, somewhat double: this may last many days, then disappear, and return again, without any decided alteration being discoverable. In one patient I remarked, that this hoarseness occurred at the menstrual periods, although the menses had ceased; on the other hand, in another patient, I did not remark this change of voice at all. On examination, we see that the nipple has become flatter and broader, and the areola surrounding it attained a great extension; the tumor at first gives a sensation of tightness, without the colour of the skin as yet having suffered any change. Later, when the breast has acquired a more remarkable size, to a superficial examination it appears softer, and it is only by a deeper pressure of the examining finger, that those hard-feeling and enlarged acini of the mammary gland are to be met with. At this period we find the veins in the surrounding skin more extended, and the breast assuming a somewhat bluish tinge, yet, without a remarkable change of colour. Thus, this disease continually advances, and according to circumstances, tolerably quickly; the affected breast acquires an immense volume, extending to a length of from 18 to 20 inches, or more, with a circumference of from 20 to 24 inches, weighs from 10 to 12lbs., and renders motion difficult to the patient. The perspiration, as well as the blood freshly drawn, has a peculiar odour, and the latter contains much free carbonic acid.* According as the swelling, through this excessive growth of the gland, advances, the rest of the person grows thinner by degrees, and by comparison, the affected breast appears to increase more rapidly in its enlargement. Later, the organs in the cavity of the thorax are forced to sympathize; now comes difficulty of breathing, with tightness in the chest; then cough, at first dry, but afterwards accompanied by frothy sputa, sometimes mingled with streaks of blood. The strength disappears, hectic fever follows, and death, from general exhaustion, sometimes preceded by the symptoms of hydrothorax. However, the further progress of the disease often differs from the picture now given, and in respect to its termination, offers many deviations; for this swelling may arrive at a certain stage of development, and there remain stationary for a series of years, without the interference of art; or, during the whole life-time of the individual, with no further disadvantage to the general organization, than what is necessarily endured from the enormous volume of the breast."

The terminations of this disorder are —1st, in recovery, which is seldom perfect, the gland never returning to its normal volume; 2ndly, in other disease, encysted alterations for example; 3rdly, in death, important organs sympathizing.

*Anatomical Details.* — The internal structure, says Dr. F., of the mammary gland is, with few exceptions, unaltered; and despite of the extensive growth in the whole, and the increase of volume of the separate acini, the latter do not exhibit any deviation from the natural structure. The cellular tissue is looser, its cells larger, and we commonly find much fat accumulated within it. The arteries do not exhibit any change, either in structure or dimension. On the other hand, the internal milk-vessels are swollen, increased in size, and the veins are always remarkably enlarged, and sometimes altered in structure. The nerves have not become smaller or thinner: yet their size compared with the magnitude of the hypertrophied gland, makes them appear diminished, although retaining their original size. Yet, where the nerves are harder and firmer, we find that the nervous mass has lost some of the medullary tissue. In the hypertrophied breast we find the growth of the mass, the increase of volume,

* ℥xvj. received in vacuo, contained 1,002 free carbonic acid. I obtained similar results from patients labouring under asthma and chronic bronchitis; on the contrary, the blood drawn from those suffering from abdominal and nervous diseases, in general, contained only a small quantity, and often no trace whatever of free carbonic acid.

and the increase of the absolute weight, and in the same proportion as the mass increases, so also does the absolute weight; it is not so with the specific weight, as is well known to occur in inflammations. With this also combines the remarkable formation of venous blood-vessels, and enlargement of the veins, whilst the arteries retain their normal size; and although we cannot give the reasons for it, yet is it nevertheless a fact, that the organs best supplied with venous blood, are those most disposed to hypertrophy; and that in the parts affected with this morbid growth, the mass of venous blood-vessels arising from enlargement of the veins, increases in the ratio of the growth of the swelling, and the increase of volume, and *vice versâ*.

*Causes.*—Puberty stands at the top of the list. Its influence may be increased by too heating food, by manipulation, and stimulating applications to the breast, by a blow, or some source of local irritation: it must be owned that the precise and efficient causes of this affection are any thing but well understood.

*Treatment.*—On this head we do not see much to detain us. Dr. Fingerhuth's hints are such as would occur to most practical men.

When the disease is incipient, derivatives, attention to the secretions, and particularly to the menstruation, occasional leechings of the feet or even of the breast, evaporating applications to the latter, and the careful removal of all likely causes of local irritation or general plethora, are means which are rational enough. Iodine or burnt sponge, or we may add, the liquor potassæ, may be administered internally, and the various ointments of iodine or mercury externally.

If the disease has gone beyond that stage which medicine can control, and if it is proceeding to an extent incompatible with life, extirpation is a severe remedy, but it is the only one.

---

### Thoracic Inflammation Masked.

Baglivi, long ago, deplored the difficulty of distinguishing, and still more of curing diseases of the lungs. Many people think that the stethoscope has removed this source of complaint; but they are mistaken. In the first place, very few, comparatively speaking, understand auscultation, even when they practise it—and, in the second place, many who have long studied the stethoscope, are liable to deception, from many causes which we need not here detail. We will illustrate these remarks by some particulars of an instructive case which we lately witnessed.

A gentleman of middle age, who had been long subject to winter-cough, and who was of a nervous temperament, was, exposed, during a journey into the country, to cold and wet, in the month of April of the present year. He returned to London very ill—his prominent complaint being incessant bilious vomiting, with cramps, spasms, and pains in the region of the stomach, but chiefly under the margins of the ribs of the left side. He had also considerable fever, but not more cough than he usually had in the spring of the year. The attention of the medical attendant was chiefly directed to the gastric irritation, and salines, opiates, and calomel were employed, with leeches and sinapisms to the region of the stomach. In the course of a few days (about four) the vomiting ceased, but the fever continued, and the spasms and pain under the false ribs of the left side persisting, the narrator was called in. The pulse was 140, and small—tongue dry and furred—thirst great—violent pain in the left side below the region of the heart—very little cough—dull sound on percussion in the lower portion of the left lung, and also in different parts of the right side—*the action of the heart could not be heard by the ear or stethoscope*—rale muqueux pretty general in the chest—skin hot — urine very scanty and high-coloured. Leeches had been applied that morning to the left side, and the bites were still bleeding freely. The danger was considered imminent—

and he died early the next morning, about fourteen or fifteen hours after the consultation. A dose of calomel and opium rendered the latter hours of existence little dolorous.

On dissection, a great portion of the left lung, especially the inferior lobe, was densely consolidated by red (recent) hepatization. The lower portion of the pleura-costalis, and all the left pleura-diaphragmatica were intensely inflamed, and a layer of recent coagulable lymph, as thick as a penny-piece, glued the lungs to the ribs and diaphragm on that side. Several portions of red hepatization were also discoverable in the right side, but no recent pleuritis.

These phenomena sufficiently account for the intense pains, spasms, and vomiting, which masked the pneumonia and pleuritis to a considerable extent, and disguised the real nature of the malady, even till within a day or two of death. It is not very easy to account for the inaudibility of the heart's action, when the ear or stethoscope was applied to the cardiac region. There was nothing abnormal found about the heart or pericardium. Some patches of incipient inflammation were found about the cardiac orifice of the stomach.

---

### Use of the Solid Nitrate of Silver for the Gonorrhœa of Women.

There is a very warm recommendation of the nitrate of silver, by Dr. Hannay, of Anderson's University, Glasgow, in the Medical Gazette.*

Gonorrhœa in women is generally said to be a very curable complaint. We cordially agree with Dr. Hannay in denying in toto that it is so. Nay we will go farther than he does; for while he admits that it is "much more so than in men," we believe that, on the whole, it is less so. The acute symptoms are pretty readily removed, it is true; but a chronic discharge is generally set up, and when set up, is almost always obstinate.

The following is Dr. Hannay's mode of applying the caustic:—

"I introduce a stick of nitrate of silver into a quill, and tie a thread firmly round the lower part of the quill to fasten the caustic, which I leave projecting beyond the quill about half an inch. I generally smear the quill with a little lard, and introduce the nitras argenti up to the os tincæ, or as far as it can be made to ascend in the vagina. I then deliberately and slowly withdraw it, turning it round so as to bring it in as extensive contact as possible with the lining membrane of the vagina. I may add, that by accident the nitrate of silver has more than once broken in the vagina, and could not be found. It caused me much alarm and anxiety at first, but after the following case I was not so affected; and though I would carefully avoid it, I now regard the occurrence as of very little importance.

The late Mr. John Herbertson, who acted as my assistant in the hospital, came in breathless haste, and in the utmost state of alarm, to call me to his assistance to extract a piece of the nitrate of silver from the vagina of a woman, who, having previously been in the hospital, and cured by the use of the remedy in question, came to Mr. H. to have it again applied. On repairing to the house we searched in vain for the caustic, but had abundant proofs that it had dissolved in the vagina. The quantity he asserted to be above two drachms. She suffered little or no pain, and a perfect cure was straightway accomplished."

It would appear that this has become a sort of party question in Glasgow. Some persons there, have denounced the practice as barbarous and dangerous. On these Dr. Hannay, and some friends of his who correspond with him, are exceedingly severe; and all parties would appear to have been infected with the subject, and to be exceedingly *caustic* towards each other. To evince Dr. Hannay's confidence, it is only necessary to quote such a passage as this:—

* Med. Gazette, May 6.

"I fearlessly give it out as an infallible and safe remedy for this disease, without any one drawback but the vain fears of persons of no experience, or of such as are determined to oppose it. I have now employed it in above 300 cases with unvarying success, and shall continue to use it."

Bona verba quæso, Gentlemen.

---

### On the Condition of the Brain in Sleep, Epilepsy, Apoplexy, &c. By Herbert Mayo, Esq.

In Mr. Mayo's recent work on the "Philosophy of Living," we met with some observations on the foregoing subjects, which have surprised us a good deal. We shall quote the passage in order to prevent any misunderstanding.

"A case is related by Blumenbach, of a person who had been trepanned, and whose brain was observed *to sink when he was asleep*, and swell out when he was awake,—*a proof that the cerebral circulation is more languid when we sleep than when we are awake*. The brain is not moved and excited by the same eddy and tumult of the blood during sleep as during the hours of waking. Accordingly, all the circumstances which hurry the circulation, such as mental contention, thought, joy, anxiety, bodily exertion, excite the heart's action, and are antidotes to sleep.

It is not equally easy to prove, but on reflection it appears no less certain, that *the nervous power of the brain is lowered in sleep;* that the depression of the cerebral circulation is accompanied by depression of cerebral energy. But how else are we to account for the slowness or suspension of digestion during sleep, the feebleness of the heart's action, the susceptibility of cold. 'The steam has been turned off,' and the body is relaxed; its functions (a fact best perhaps shown in hybernating animals,) are half at a stand-still; that which imparts force and activity to every function, is no longer generated in adequate power and quantity; the brain, the source of nervous energy, is in repose.

The bearing of these conclusions, if just, upon cerebral disease, is of supreme importance. *Brain attacks generally come on during the night, and during sleep.* That is to say, *they mostly supervene at the time when the power of the brain is lowered.* They are then, in some degree, connected with depression of the cerebral forces. They are favoured by weakness and exhaustion of the brain.

The attacks to which I refer are epilepsy, apoplexy, palsy; the common impression respecting which is, that they proceed from determination of blood to the brain, or from some kind of force or pressure operating actively to disturb the functions of the organ. I believe, on the contrary, that in the majority of cases, especially in advanced life, all these seizures result from cerebral failure, from weakness, depression of power, temporary or permanent, of some part or the whole of the brain.

It does not contravene the preceding conclusion, that these complaints are liable to be primarily induced by *action in the head;*—that where they do not result from alteration of structure, they often may be traced to habits of full living and strong excitement, which have frequently thrown the blood in hurried and violent circulation through the brain;—and that besides, in many instances, a loaded and laboured circulation goes with, and gives increased danger to such attacks;—and that nothing is more likely to benefit *the latter class of cases* than diminution by means of cupping, of the quantity of blood in the vessels. But grouping together all cerebral seizures that take the form of fits, I believe that the cases in which cerebral congestion is a feature, *are the exceptions;* and that it is most important the practice grounded on this principle should be recognized, that *diminishing the quantity of the blood is not the appropriate remedy for cerebral seizures.*"

In the first place, a man with half the science which Mr. Mayo possesses, must be aware that a brain, when the skull is trepanned, is not at all in the physiological condition of one where the cranium is imperforate. Can it be supposed that the brain heaves and retreats, dilates and contracts in volume when the atmosphere is excluded? If it do, what supplies the place of the diminished size of the encephalon? We hold it to be physically impossible that the brain is smaller in size at one part of the day than at another. Then, as to the "slowness or suspension of *digestion* during sleep," we know, from observation and experience, that digestion goes on more quickly during rest and sleep, than during exercise and waking;—in short, that the proposition above-mentioned is just the reverse of fact.

In respect to the "*feebleness* of the heart's action" in sleep, we join issue with Mr. Mayo, and aver that, in sleep, the heart beats *fuller* and slower than when the individual is awake, and that the circulation goes on more uniformly and freely over the whole surface of the body in the former than in the latter state. Who knows not that the face is redder, the features more expanded, the surface warmer, and the capillary vessels fuller in sleep than in waking? The analogy between sleep and hybernation is a very forced and false one. The functions are not "half at a stand-still" in sleep. They go on—secretion, absorption, assimilation—every function except those appertaining to sense and intellect, just as well when we are in the most profound sleep, as if we were all alive to the most vivid sensations and reflections—perhaps better.

Then as to "brain attacks" coming on more frequently in the night than in the day, we are ready to grant the proposition, but deny the inference which Mr. Mayo has drawn from it. It is not because the "power of the brain is lowered," but because the horizontal posture and the fuller circulation combine with a loaded stomach to *oppress* the brain, either in consequence of greater impetus of blood *to* that organ, or obstruction (for obvious reasons) to its return from the head.

Finally, we most strongly protest against the doctrine of taking fulness of the cerebral vessels, in "fits," as the *exception*, and inanition as the general rule. A more dangerous precept was never laid down for the guidance of young practitioners—and, à fortiori, for the instruction of popular readers. If acted on, it would annually prove the destruction of thousands. Is it only in the *exceptions*, that we find the brain torn up in apoplexy, or the vessels gorged as if injected with red wax? In epilepsy, the disease generally begins in youth, or in middle age at farthest; and are those the periods for emptiness of the cerebral vessels, or diminution of energy in the brain? No indeed! We would advise Mr. Mayo to modify these statements in his next edition, otherwise they will be productive of much mischief. In anatomy, physiology, and surgery, no authority is better than that of Mr. Mayo—but as to the details of practical medicine, we would advise him to bear in mind the homely but wholesome adage—"ne sutor." Verbum sat.

---

### VITAL STATISTICS.

### 1. *Value of Life in Geneva.*

Our contemporary, the Medical Gazette quotes some passages from an article on vital statistics, published by M. E. Mallet, in Annales d'Hygiene Publique. The subject of the paper is the population of Geneva from the year 1549 to 1833.

The following are the statements of M. Mallet on the mortality of different ages:—

The first day of life is also the day of death to so many as one in fifty-one. The second day is three times less fatal than the first. On the third, fewer by one-half die than on the second. The mortality diminishes further on the following days, regularly, but less rapidly. The remainder of the first month is, however, still very dangerous, since the death of about half the children who die in the first year takes place in this first month, which is thus eleven times more destructive than the others. The deaths during the first month amount to 6·85 per cent. of the births.

The mortality diminishes from four and a half to one, from the first to the second month; from two to one from the second to the third; from three to two from the third to the sixth. In the last six months of the first year the diminution of mortality is scarcely perceptible. During the whole first year one infant dies out of 7·2.

The second year is three times less dangerous than the first; it carries off one in twenty-one. The third is still less destructive by one half, taking one in forty-two.

From three to eight years the mortality decreases nearly two-thirds. From eight to seventeen it is very feeble; it is during this period that the years of lowest mortality occur, the tenth and the fourteenth. After seventeen years the proportion of deaths increases about a third, and remains balancing about the same rate till about forty-five. From that age it increases, at first gradually, but soon at a more rapid rate. Thus from fifty-one to sixty death carries off a fourth; almost half of those who attain the age of sixty-one die before they attain seventy. Of septuagenarians three-fourths fall before eighty; and ten-elevenths of those who pass eighty die before ninety. The total number of those who have completed ninety years is fifty-six, or one in 194 (of the last class, we presume.) The oldest of these died at ninety-nine, no example having occurred of an individual at Geneva living a hundred years.

The Genevese of the present age were surpassed by their ancestors in fecundity, and surpass them in longevity. Our contemporary observes that this is a sure proof that the modern Genevese are much wiser than their forefathers—and we agree with him.

2. *Abuses of Vital Statistics.*

Our readers must be aware of the value we attach to the prosecution of statistical researches. And they must be aware also, that we have laughed now and then at the extravagant anticipations of some of the statisticians. Mr. Edmonds, for example, occasionally advances some startling propositions, and one or two statistical writers in this country would induce us, if we would allow them, to fall back on the médecine expectante, and treat our patients on the "average" system.

The Royal Academy of Medicine at Paris has lately been addressed on this subject by M. Double, who has been at some pains to point out the inapplicability of statistics to the actual practice of medicine. We subjoin the following observations of M. Double, from an account of his paper contained in the Medical Gazette.*

" In mathematical analysis, the probability of future events is calculated from the observation of preceding facts, but always under the rules of the universal laws of large numbers, and without any individual application.

In medical statistics, on the other hand, the numerical method is expected to determine from the observation of preceding facts, and according to their number, the best method of treatment in each individual case which may occur. This, however, is quite impossible; and I may remark, that were it ever effected, medicine would cease to be either a science, an art, or even a profession: it would become as mechanical as the employment of the shoemaker.

What is called in geometry the universal law of large numbers, is the rule and the foundation of all calculations of probabilities. One of the conditions of this law is, that the causes of the events calculated, though some are constant and others variable, yet can in no sense be said to vary progressively. From this law it results, that all the differences and irregularities which balance each other disappear in the quotient: and in this way the calculations of lotteries, of maritime insurances, &c. are made.

But this is evidently not applicable to medicine: neither our successes nor our failures balance themselves in large numbers, as in the case of marine insurances. Each of our problems embraces but one individual; and besides, diseases always have their prevailing character, varying progressively according to an infinite variety of causes."

* June 3, 1837.

"The numerical method at once supposes and sanctions one of the greatest errors in therapeutics,—namely, the adoption of absolute and exclusive measures. The celebrated problem of Pickairn—'For a given disease to find the remedy'—is only reasonable, when understood in this way—'For a given indication, to find the best method of fulfilling it.' Each individual malady is not a simple phenomenon that can be represented by unity: it is not certain and fixed, but constantly varying. Thus the pneumony of to-day is not the pneumony of yesterday, and the pneumony of Peter is not that of Paul."

While we agree in many respects with M. Double, we cannot go the whole way with him. It is very true that each case is a separate problem, or, in other words, that no two cases are *exactly* similar. But it does not therefore follow that no two cases are *so* similar, as not to require the same general mode of treatment, and to admit of a fair comparison of results with two other cases treated in another way. This is riding the eclectic hobby over hard. Common sense existed before statistics, and common sense has practically decided against M. Double. What is it but a species of numerical calculation, that has determined that bark is a better remedy for ague in the gross than bleeding is? What is it but a species of statistics that has settled all our great therapeutical opinions and methods? Diseases admit of the application of statistics to their treatment, provided that application be not too rigid. It is true, as M. Double observes, that it would be insanity to treat every individual case by some unyielding law deduced from previous numerical results. No one, we should hope, would contend for that. But the treatment of the individual cases will be greatly directed by those results, though a well-informed physician will allow the general principles of treatment to be modified by the special phenomena.

---

### THE BRUIT DU DIABLE.

The *bruits* are multiplying most alarmingly. We know not exactly how many there now are of them, but they would constitute a gamut, and honest plain folks are beginning to despair of ever being able to master them. It would be interesting to ascertain whether the correctness of diagnoses has augmented in the same ratio as the number of the bruits, and the minutiæ of auscultators. For our own parts, we are rather inclined to doubt it. But to the point.

M. Bouillaud has described a "bruit du diable," so called because it resembles the sound of a humming top, or "diable." He has heard it often in anæmic, chlorotic, and nervous subjects. He says that it rises and falls according to the pressure on the vessel, and the activity of the pulse, as the tone of the instrument varies with the strokes of the whip which urges it. It is chiefly heard over the course of the left carotid and subclavian, above the inner end of the clavicle. It is subject to sudden intermissions, for which M. B. is unable to account; and it is weakened or stopped entirely by a pressure on the arteries not sufficient to arrest the pulse. It ceases also if the artery be compressed above the place where the stethoscope is fixed, or if the larynx be pushed aside from that part, and also during a violent effort; but it is much augmented in intensity if the head be turned away, and the chin be raised. M. B. considers the *ronflement du diable* to be a sound intermediate between the bellows murmur and the musical sound of the arteries described by Laennec, and says that it is attended with a purring tremor of the parietes of the chest, of a slighter character than that which indicates contraction of the cardiac orifices. The same class of persons—viz. the nervous and the chlorotic, were the subjects of one or other of these sounds; and so constantly does the "*bruit du diable*" attend chlorosis, that he terms it the "*bruit chlorotique.*" He has also observed it in pale nervous men, but he does not decidedly state whether it was this sound or the others that he heard. He attributes it partly to a watery state of the blood, as it ceases to be audible with the return of health, and the restoration of that fluid to its normal condition; partly to the rapid circulation so near the heart; and in part to the vicinity of the larynx, which acts as a kind of sounding-board.

Such is the condensed account of M. Bouillaud's observations, presented by Dr. Ogier Ward, in the Medical Gazette. He furnishes it, in order that he may offer a theory of his own to explain the phenomenon in question—a theory which places the cause of the sound solely in the veins. His reasons are the following:—

"First, the sound has been heard over the course of the external jugular vein as well as over the carotid arteries. Secondly, it is solely in this latter situation, and not always there, that the sound is so modified by the proximity of the carotid as to appear to be augmented by the ventricular systole. Thirdly, it is arrested by pressure on the external jugular when heard along the course of that vessel, and, as is asserted by M. B. by a pressure over the carotid quite insufficient to stop the pulsation of the artery, but which would certainly obstruct the flow of blood through the internal jugular. Fourthly, it is increased by pressure, and by everting the head with the chin raised; because, under these circumstances, the calibre of the vein is diminished, and consequently the velocity of the current is increased. Sixthly, for the contrary reason the sound is stopped by turning the head so as to shorten the vein, and retard the course of its contents towards the heart. The greater distance the blood has to traverse in passing from the left side of the neck into the vena cava, will also explain the greater frequency of the sound on that side. Seventhly, the sound is arrested by pushing aside the larynx, because this can be accomplished only while the muscles of the fore parts of the neck are relaxed, at which time the jugular vein is relaxed also; besides that, the larynx and trachea sometimes prolong, by their resonance, the pulsations of the carotid and the respiratory murmur, so as to simulate the bruit-du-diable when none exists. Eighthly, a violent or a prolonged effort produces regurgitation in the veins, and thus puts a stop to the flow and sound of their contents. When the sound intermits, I have been able to account for it by the pressure of the stethoscope, or by the position of the patient's head. Tenthly, the reason that the purring tremor, when felt, is slighter in these cases than when a contraction of the cardiac orifices exists, arises from the difference in the strength and tension of the vessels, by whose vibrations the phenomenon is produced."

Dr. Ogier Ward concurs with M. Bouillaud in referring the bruit du diable to a too fluid state of the blood, with increased rapidity of the circulation. Of five patients whom he has seen with it, four have been affected with palpitations, and two have been chlorotic.

---

### Chemical Constituents of Calamine.*

The powder sold in the shops under this name, and continually used by surgeons, consists, if the analysis of Mr. R. H. Brett be true, of—

Sulphate of barytes!
Oxyde of iron.
Carbonate of lime.
Lead (probably sulphuret)
Zinc? (mere traces.)

---

### Seven Half-crowns Swallowed and Voided after Twenty Months.

The case is reported by Mr. Wakefield, surgeon to the House of Correction.

"Twenty months ago, a man of the name of Seeley was sentenced to three years' imprisonment in the House of Correction. On the day of his admission, (under the apprehension, I believe, of the money he had in his possession being taken from him,) he swallowed seven half-crowns. Shortly afterwards, alarmed at his rash act, he mentioned the circumstance to one of the turnkeys, who, however, gave no credit to the tale; and in a few days, feeling no inconvenience, and concluding the pieces of money had passed with his motions,

* Med. Gaz. April, 15.

the affair was forgotten. About a fortnight ago this man complained to me of sickness and slight bowel-complaint, with general tenderness over the abdomen; for which he was removed into the Infirmary, and I prescribed some small doses of mercurial medicine, with Dover's powder, on the fifth day; and while under this treatment, to his great astonishment, the seven half-crowns fell clattering in one motion into the close-stool pan, having been retained in the intestinal canal upwards of twenty calendar months. Their colour was perfectly black; but upon examining them very carefully no loss of substance could be discovered."

### COUGH ENDING IN EMPHYSEMA OF THE BODY.

Mr. F. G. Hicks, a young surgeon of much information and ability, has published an interesting case of this description.*

The patient was an infant, ten months old, who had laboured under cough for some days prior to the 21st of March, when Mr. Hicks first saw him. The cough was then violent, spasmodic, and occurred in paroxysms. Appropriate means were used, but the complaint was not relieved, and on the 30th, drowsiness, slight strabismus, and other symptoms of cerebral affection came on. By calomel, cupping, &c. these symptoms were relieved, but the cough continued, and in the evening of the 2nd of April, Mr. Hicks was sent for. The child had had two or three severe fits of coughing that day, and Mr. Hicks found extensive emphysema of the chest, neck, and abdomen, with very laborious respiration. Next day the child died.

*Dissection.* There was extensive emphysematous swelling about the neck and chest, and extending over the abdomen and down the thighs. On raising the sternum with the cartilages of the ribs, several large bags of air presented themselves, which were found to be formed by air having got between the two layers of the anterior mediastinum. The whole of the cellular membrane in the anterior of the chest was filled with air—more especially on the right side, and at the root of the upper lobe of the right lung. At this point, when the lungs were inflated by means of bellows, the air appeared to escape under the pleura, and here it was evident the lung had given way, causing this extensive emphysema. The lungs were much collapsed, especially the left, the right being somewhat emphysematous. They were much tuberculated, especially about their apices. The abdominal viscera were healthy. The head was not examined.

* Med. Gaz. April 22.

### DIABETES.

1. ON THE COMPARATIVE STATE OF UREA IN HEALTHY AND DISEASED URINE, AND THE SEAT OF THE FORMATION OF SUGAR IN DIABETES MELLITUS. By R. M'GREGOR, Esq. &c.*
2. CASE OF DIABETES MELLITUS. By HENRY SNOWDEN, Esq. of Hull.†
3. MR. LEACH ON LYTTA IN DIABETES.

I.—Mr. M'Gregor is lecturer on Chemistry to the Portland Street Medical School in Glasgow, and was formerly apothecary to the Glasgow Royal Infirmary. He has contributed a long and an interesting paper to our contemporary, the Medical Gazette, on the composition of diabetic urine. The paper in question contains several experiments. These are too long for us to quote, but their results admit of being thus summed up:—

1st. "That we know of no disease characterised by the absence of urea; though in some the quantity of it is, in common with some of the other ingredients, diminished.

* Med. Gazette, May 13—20.

† Ibid. April 22.

2nd. That the quantity of urea is quite independent of that of the sugar; the former increases, not by reason of the disappearance of the latter, but because the patient has been put upon liberal doses of animal diet, opium, or some other powerful palliative, which checks the excessive thirst, and thus concentrates the urine. In the case of the healthy individual who was fed upon animal food and water exclusively for three days, the quantity of urea passed more than doubled the natural diurnal amount, shewing that large quantities of animal food increase the urea proportionally. Opium has a similar effect, both in health and diabetes. In the diabetic individual, who was similarly treated for three days, the urea crystallized readily on the addition of nitric acid, notwithstanding the presence of a large quantity of sugar.

3. That in diabetes, the quantity of urea is materially increased in consequence of the greater quantity of food introduced into the stomach, either vegetable or animal.

In the urine of herbivorous animals, we have a goodly proportion of urea. In one specimen, of that of a cow, I found 3.2 per cent. of urea.

4. That the sugar is formed in the digestive organs, and that the kidney is a mere outlet in common with other excretory organs of the body.

And, lastly, that it is readily discoverable in the blood, saliva, and stool of diabetic patients, and even in the blood of healthy persons who indulge in vegetables."

II. Mr. Snowden, of Hull, has related a case of diabetes insipidus, successfully, or apparently successfully treated by diuretics.

The gist of Mr. Snowden's observations is this :—*that*, the only constant morbid appearance in fatal cases of diabetes, is enlargement and turgidity of the capillaries of the kidneys—*that* this condition is essentially one of atony—*that*, it is highly favourable to secretion and exudation—*that*, it is remediable by the application of stimulants—and *that*, therefore, diuretics are indicated in diabetes.

A patient, aged 17, who had the usual symptoms of diabetes (nothing is said of its being the saccharine form, a material point), and who for ten months had voided every twenty-four hours, from five to twenty pints of urine, became the subject of experiment. When admitted he passed sixteen pints in the time mentioned, the quantity of fluid drunk being ten pints. The specific gravity of the urine was 1004. Analytical examination of 1000 grains gave water 987, urea 9.5, salts 3.2, a trace of uric acid, the rest lactates, with animal matter.

Venesection, diaphoretics, tonics, astringents, animal diet were employed without success. On the 10th of February, 1837, he passed twenty pints of urine. He was then ordered—

Habeat solutionem Potass. Bitart. pro potu ordinario.

℞ Potassæ Nitrat. ℈iv; Aquæ, ℥viij. capt. coch. ij. ter in die.

℞ Spt. Ætheris Nitrici, ʒj. ; Aquæ, ℥j. ; M. ft. haust. omne nocte sumendus.

This treatment was continued till the 2nd of March, when he passed six pints of urine. The medicine was then changed for infusion of quassia and spir. æth. nitrici. On the 16th of March, he was discharged cured, two issues being on that day established over the region of the kidneys. He passed three pints and a half of urine.

This case is dated the eleventh of April. It needs but a small acquaintance with diabetes, to be aware, that many cases have seemingly been cured, on as good grounds as the preceding, yet have in the end proved fatal.

When we turn from Mr. Snowden's fact to his reasoning, we fear there are some grounds for hesitation. In the first place, it is probable that the enlargement of the renal capillaries is an effect of the over-work of the organ, rather than a cause of the diabetes. In the second place, it is not certain that, granting such enlargement to constitute the disease, diuretics would tend to diminish it. It is very true, that passive inflammation of a mucous membrane is not unfrequently relieved or removed by the direct application of a stimulant. Conjunctivitis, for instance, may be cured by a lotion of nitrate of silver. But diuretics cannot be considered as stimulants to the kidney of an analogous description. They rather urge the whole organ to increased exertion of its function, than act as mere local stimulating applications. But we shall be glad to see the experiment repeated by Mr. Snowden, or others.

III. Mr. Leach, of Heywood, Lancashire, is sanguine. He has written a letter in the Gazette,* in which he warmly encourages the idea that Mr. Snowden's recommendations may prove of great service. Nay, he has acted on them in one case of diabetes mellitus, and, apparently, with benefit. But although the patient improved more than once, he died of the disease, a circumstance attributed by Mr. Leach to his intemperance, which counteracted the good effects of the remedy.

The tinctura lyttæ we know to be useful in cases of enuresis. Whether it will cure diabetes is another matter. We own that we are sceptical.

---

The Northern Flora, or Description of the Wild Plants of the North and East of Scotland, &c. By Alexander Murray, M.D. Part I. Edinburgh, 1836.

Dr. Murray is a resident physician at Aberdeen, and seems to be not only a very zealous, but also a very excellent, botanist. This first part of his catalogue of the indigenous plants found in the North and East districts of Scotland, is, in our opinion, quite a model of what a Flora ought to be. It is written throughout in English, and thus avoids all the ambiguity and pedantic uncertainty of Latin descriptions, which used formerly to tease us exceedingly in our botanical enquiries.

The excellent work of Dr. Greville, the Flora Edinensis, is upon the same plan, and has for many years contributed most essentially to diffuse a taste for these delightful pursuits among the medical students at the Edinburgh University.

On the rambler among the Highlands of Scotland Dr. Murray has conferred a most useful and agreeable present; and we quite trust that he will meet with such encouragement, as will induce him to complete the task, which he has so very ably commenced. The present part embraces the first five classes of the Linnean system.

In the preface Dr. Murray acknowledges the assistance of many friends, who have communicated the *habitats* of the plants described. Among these we observe the names of the Earl of Aberdeen, to whom the work is dedicated, and of Dr. Graham, the Professor of Botany at Edinburgh. The valuable contributions of the latter to the Edinb. New Philosophical Journal have gained for him the well-merited thanks of every lover of practical botany.

---

Tic Douloureux relieved by Galvanism.

Mr. Grantham, of Crayford, Kent, has reported this case, with some others, in the Medical Gazette.

*Case.* A tailor, aged 47, was attacked in the summer of 1829, with tic douloureux, the right facial nerve being the one affected, attended with involuntary contraction of the temporal, pterygoid, buccinator, and levator anguli oris, muscles. These paroxysms returned at intervals with increasing severity, sometimes lasting fourteen days without intermission. The removal of the teeth, salivation, arsenic, carbonate of iron, &c., were all productive of benefit, which, however, was but temporary. He would seem to be cured for three or four months, and then the complaint would return as badly as ever. In the beginning of July, 1836, Mr. Grantham tried a galvanic battery of 40 single plates, three inches square, each pair connected at the upper part with copper-wire, resembling Dr. Wollaston's battery,—the *Couronne des Tasses* arrangement. The patient was at this time severely affected. Shocks

* May 13, 1837.

were passed from the back part of the head, both sides of the face, down the neck, in the direction of every principal nerve, but more generally from the region of the parotid gland to the exit of the inferior maxillary nerve near the chin. The shocks from this battery were very severe—so powerful, indeed, that few could have borne them. Where the shilling which communicated with the wire touched the skin, it caused excoriation of the cuticle, till at last the pain of the shocks exceeded in severity the pain of tic douloureux, especially over the region of the parotid gland. During this process, he took carbonate of soda twice a day, in two-drachm doses.

September 12th, 1836, he felt better than ever he had done; since which time he has remained well, being enabled to wash his face with cold water, which he has not done before for the last three years.

We must not "holloa before we are out of the wood." The patient is relieved, but it is not certain that he is cured.

---

### Substance of a Statistical Report from the Morgue. Paris, 1836.*

The abridged statistical report, of which this is an additional abridgment, has been communicated to the Lancet by Dr. O'Reilly, of Dublin.

It appears that, in 1836, there were received at the Morgue:—311 bodies, or portions of bodies.—279 of different ages: males, 235; females, 44. In the 279 we have,—

Male suicides, 80; female, 28; suicides not proven male, 58; not proven female, 6; 172 probable suicides.

Male, homicide, 1; female, 3: 4 homicides.

Accidents, male, 52; females, 4.—56 accidents.

Sudden deaths, male, 44; female, 3. 47 natural, or sudden, deaths.

Males, 235; females, 44. Total, 279.

*Nature of the Death.*

Submersions, males, 151; females, 28; 179 submersions.

Sudden deaths, males, 39; females, 3. 42 sudden deaths.

Deaths from fire-arms, males, 12; females, 1. 13 from fire-arms.

Asphyxia from charcoal, males, 5; females, 4. 9 asphyxiated by charcoal.

Falls from elevated situations, males, 4; females, 2. 6 from falls from elevated places.

Suspensions, males, 6; females, 0. 6 from suspensions.

General burning, male, 1; female, 1. 1 from extensive burning.

Crushed under carriages, males, 9; females, 2; 11 crushed by carriages.

Sudden deaths, males, 5; females, 0. 5 from diseases.

Homicides, male, 1; females, 3. 4 homicides.

Poisoning by the inhalation of noxious vapours, 1, from mephitism.

Poisoning, males, 0; females, 1. 1 from poisoning.

Sharp, or cutting, instrument, males, 1; females, 0. 1 cutting instrument.

Number received in each month, male and female,—Total, 279. The maximum number was in May, and the minimum in November:—

| | | | |
|---|---|---|---|
| May...... | 41 | December .. | 22 |
| March.... | 30 | February ... | 19 |
| July...... | 29 | September.. | 18 |
| April ..... | 27 | October .... | 18 |
| June...... | 26 | January.... | 13 |
| August.... | 25 | November .. | 11 |

Table of the ages of the individuals carried to the Morgue.

| | Males. | Females | Total. |
|---|---|---|---|
| From 5 to 10 | 2 | — | 2 |
| 10 to 15 | 6 | 1 | 7 |
| 15 to 20 | 14 | 3 | 17 |
| 20 to 25 | 16 | 4 | 20 |
| 25 to 30 | 28 | 4 | 32 |
| 30 to 35 | 23 | 3 | 26 |
| 35 to 40 | 24 | 2 | 26 |
| 40 to 45 | 19 | 7 | 26 |
| 45 to 50 | 22 | 3 | 25 |
| 50 to 55 | 21 | 3 | 24 |
| 55 to 60 | 15 | 3 | 18 |
| 60 to 65 | 15 | 6 | 21 |
| 65 to 70 | 12 | 1 | 13 |
| 70 to 75 | 7 | 1 | 8 |
| 75 to 80 | 5 | — | 5 |
| 80 to 85 | 2 | 1 | 3 |
| Age not determined.......... | | | 1 |

The suicides recognized were,—mar-

* Lancet, June 3, 1837.

ried, 33; unmarried, 45; widowers, 17; widows, 5. The professions of the suicides were,—

| | | | |
|---|---|---|---|
| Military | 10 | Labourer | 1 |
| Invalids | 2 | Gardener | 1 |
| Old soldiers | 1 | News-venders | 5 |
| Military workers, | 1 | Postilion | 1 |
| Joiners | 3 | Hosier's clerk | 1 |
| Printers | 2 | Librarian | 1 |
| Tailors | 2 | Plasterer | 1 |
| Wine-merchants | 2 | House-holder | 1 |
| Shoemakers | 2 | Knife-grinder | 1 |
| Masons | 2 | Skopkeeper | 1 |
| Butchers | 2 | Apothecary | 1 |
| Pastrycooks | 2 | Coachmaker | 1 |
| Makers of cordials | 2 | Physician | 1 |
| Bakers | 2 | A registrar | 1 |
| Copper-founders | 2 | Hairdresser | 2 |
| Commission-agents | 2 | Indian-ink-maker, | 1 |
| Clerk to wine-merchant | 1 | Rope-maker | 1 |
| | | Delft-seller | 1 |
| Porter | 1 | Landladies | 4 |
| Broker | 1 | Laundry-maids | 4 |
| Jeweller | 1 | Cooks | 2 |
| Cutler | 1 | No profession | 2 |
| Paper-maker | 1 | Paper-worker | 1 |
| Stone-setter | 1 | Sempstress | 1 |
| Bell-maker | 1 | Clergyman | 1 |
| Paviour | 1 | Grocer | 1 |
| Painter | 1 | Painter in porce- | 1 |
| Bricklayer | 1 | lain | 1 |
| Varnisher | 1 | Brass-worker | 1 |
| Sailor | 1 | Unknown | 2 |

Causes of suicide which were identified:—

| | Male | Fem. | Total |
|---|---|---|---|
| Mental alienation | 10 | 12 | 22 |
| Bad conduct | 8 | 1 | 9 |
| Disgust of life | 6 | — | 6 |
| Misery | 4 | 1 | 5 |
| Robbers | 4 | 1 | 5 |
| Disappointed love | 3 | 1 | 4 |
| Loss of money | 3 | 1 | 4 |
| Quarrels and domestic disappointments | 1 | 3 | 4 |
| Loss at play | 3 | — | 3 |
| Drunkenness | 3 | — | 3 |
| Result of quarrels | 3 | — | 3 |
| Incurable disease | 2 | 1 | 3 |
| Departure from, or separation of, family | 1 | 1 | 2 |
| Brain fever | 2 | — | 2 |
| Loss of wife | 1 | — | 1 |
| Embarrassed affairs | 1 | — | 1 |
| Result of remonstrance | 1 | — | 1 |

Accidents leading to the submersion:—

| | |
|---|---|
| Bathing | 13 |
| Drunkenness | 8 |
| Boating, or sailing | 6 |
| Falling from horses, while drinking | 3 |
| Playing on the shore | 2 |
| Fishing | 2 |
| Leaping near the river | 1 |
| Washing feet, saving others, storm blowing individual into water, &c. | 4 |

On the whole, the greater tendency of the male sex than of the female in Paris, in 1836, to violent death is remarkable. It will even be observed that for one female who committed suicide for love, three males did so—a circumstance much opposed to the common notions on the subject.

---

### VARIETIES OF THE LOBELIA INFLATA, DEPENDENT ON SOIL.*

"On a wet, clayey soil the lobelia inflata is a powerful poison, its narcotic power being then in great excess, and producing, in ordinary doses, the most alarming symptoms, continued vomiting, tremors, cold sweats, and death. The greatest value of the plant is in its administration in asthma, when grown on soils which develop its antispasmodic qualities in an eminent degree. Exposure to light deprives the tincture, or any of its preparation, of its antispasmodic power, whilst the narcotic remains. Great attention should be paid, in collecting the plant, to the proper locality, and also to the elaboration of the sap. It should then be dried in a dark room, tightly compressed in bundles, and kept in air-tight boxes, in order to maintain its peculiar properties. When thus prepared, and after keeping for years, its narcotic powers become weakened, but the antispasmodic qualities are retained.

It is also emetic, expectorant, sudorific, diuretic, and sialagogous. The Indians have used it for centuries, combined with the eupatorium perfoliatum, and ilex cassina, as one of their best emetics for cleansing the stomach and head previous to holding their great councils."

So says Mr. Whitlaw, who, about 30 years ago, was in America, and took an interest in the matter.

---

* Lancet, May 20.

On Wounds of the Heart. By Mr. Lees.*

Mr. Lees has collected from various quarters, and narrated from his own observation, many cases, illustrative of the effects of wounds of the heart. In this country, at this time, such wounds must chiefly interest us in their medico-legal relations. We shall content ourselves with a summary of the more striking facts.

1. Wounds of the heart are popularly considered to be instant death. They are not always instantaneously fatal, but in too many instances they are so. Take a case as a sample.

*Case.* Dr. Corrigan shewed Mr. Lees a preparation in his possession, of a wound of the right ventricle which was followed by instant death: the case was that of a bailiff who was stabbed in the belly with a long knife, by a person standing upon the stairs under him; the knife penetrated the diaphragm just below the seventh rib, entered the abdomen, and re-entered the diaphragm, and perforated the right ventricle of the heart near the base.

2. The most interesting facts are those which establish the possibility of the individual's living for a short or a long time after the infliction of the wound. Mr. Lees cites some instances of this description.

*Case* 1. Ambrose Parè states he witnessed a case where a person, after being wounded by a sword thrust in the chest, pursued his adversary for two hundred paces, and then fell dead; on examination he found a wound in the heart large enough to receive his finger, and a large quantity of blood effused on the diaphragm.

*Case* 2. Hennen, in his Principles of Military Surgery, quotes a highly interesting case of a soldier, who received a sword wound in the chest, and was left on an open staircase for five days, during severe winter time; he lived ten days after being brought to hospital, and died of gangrene of the legs, caused by the extreme cold to which he had been exposed; on dissection it was found that the right lung had been wounded, the right ventricle of the heart laid open, but this wound was perfectly cicatrized.

*Cases* 3, 4, 5. Dupuytren was decidedly of opinion, that these wounds were necessarily fatal; and in his Leçons Orales he alludes particularly to the case of a soldier, in whom, six years after the healing of a wound of the chest, a musket ball was found impacted in the substance of the right ventricle of the heart near its apex.

He also gives the case of a man, thirty-four years of age, who was brought to the Hôtel Dieu, May 5th, 1831, having received two stabs of a knife, one in the præcordial, the other in the epigastric region; there was no evidence that the wound had penetrated, as the heart's action and respiration were regular, the patient complained but slightly, and auscultation and percussion discovered no morbid phenomena. He had, however, a general spasmodic trembling. He died on the 13th with symptoms of diseased brain, having been affected with tetanic spasms for two or three days previous to his death; there was a wound of the pericardium of three lines, which also penetrated the cavity of the left ventricle, the pericardium contained ℥i. of serosity, and Dupuytren thought he might have survived if the head-affection had not supervened.

He also gives another case of a man who died mad, after attempting suicide by amputating the penis, and in whom six penetrating wounds of the right ventricle were found after death, although during life there were no symptoms indicative of such a lesion.

*Case* 6. In the Museum at Martinique is preserved a heart, with the end of a sword five inches in length impacted in it. The case was that of an officer, who in a duel received a sword wound in the right side of the chest, the point of the weapon broke, and the

* Dublin Journ. May, 1837.

seconds supposing it to have been lost in the grass, walked with him to the hospital, where he expressed so little uneasiness, that the surgeon, supposing it to be a mere flesh wound, allowed him to return on board his ship, where he continued without any suffering the whole of that day, but at night very severe symptoms supervened, and he died next day; on examination, it was found that the point of the sword had passed through the right auricle, and wounded the left lung.

The preceding case is cited in illustration of Harvey's proposition on the insensibility of the heart—a proposition supported by his celebrated case of the young nobleman. Mr. Lees quotes also the experiments of Mr. Carlile, Demonstrator of Anatomy at Trinity College.

"He mentioned to me that in his experiments on the motions and sounds of this viscus, he found that punctures of its substance affected its motions but slightly, and even on inserting tubes through its parietes into the cavities, though at first there was some slight palpitation, yet it soon accommodated itself to their presence; but he found that if he compressed the nerves going to its substance, by passing a ligature round the aorta and pulmonary artery, much suffering was evinced."

3. Mr. Lees divides wounds of the heart into punctured and contused.

The former he subdivides into simple acupuncturation—and punctures complicated either with a copious hæmorrhage, or with residence of a foreign body in the wound.

A. Of the first of these complications, the late Duc de Berri (who was assassinated at Paris in the year 1820) afforded a remarkable examble. He was stabbed in the right side, the right ventricle was pierced through, and the hæmorrhage into the cavity of the pleura was so considerable, as to oblige Dupuytren to open the wound every two hours while life lasted, in order to prevent impending suffocation; he lived in this state for seven hours.

B. "The second complication may be caused, either by a fractured portion of a rib, or by a part of the weapon remaining in the wound. In the museum at Park-street, Mr. Thomas Hart had the kidness to show me a preparation of wound of the right auricle; and Mr. Wilkin, who was clinical resident in Stevens' Hospital at the time it occurred, has given me the particulars of the case. It was that of a brewer's man, who had fallen under a dray, when it was heavily laden, which passed over his chest; he was lifted up, and complained of pain and weakness, but was able to continue sitting on the side of the dray driving the horse for nearly an hour, when being in the vicinity of the hospital, he thought he might as well get himself examined: he walked in, and lay on a bed, but on turning on his side he suddenly expired. On dissection, it was found that the fifth rib was fractured, and that the extremity of one portion had penetrated the pericardium, but had freed itself from the heart; and this, as Mr. Wilkin observes, accounts for the sudden death of the man. For it is probable, that the portion of rib had filled up the wound of the heart, and thus prevented any hæmorrhage until his arrival at the hospital; when, on its coming out, the sudden effusion of blood into the pericardium caused sudden death; there had no blood escaped outside the pericardium."

"As to those cases, in which part of the weapon remains in the wound, Orfila quotes an interesting observation in his Medicine Legale, of a workman who, in a melancholic mood, stabbed himself with a sharp stilet, between the fifth and sixth ribs of the left side, on the 24th of May. He was brought to hospital on the 26th, in a state of great collapse, the pulse small, intermitting, respiration hurried, great anxiety, and severe pain felt; on touching the wound, which was nearly cicatrized, but just below it, a peculiar thrill could be heard, or as it is expressively denominated a *crepitation onduleure*, similar to what can be heard in a varicose aneurism: the horizontal position caused great pain. On the 3rd of June he had severe rigors, followed by erysipelas of the face, and he died on the 13th, that is, twenty days after the receipt of the wound; on examination of the body, the pericardium was found to contain ten ounces of fetid bloody serum; in the inferior third of the right ventricle was impacted an iron stilet, which, penetrating the septum, could be felt in the left ventricle."

c. The heart occasionally suffers from contusions of the thorax. In the last siege of Antwerp, by the French, some remarkable cases occurred in which this organ was severely contused, and ruptured without any external appearances of injury, either to the integuments or ribs, in which the death, in some cases instantaneous, was supposed to have been caused by the wind of the bullet. In some of the cases mentioned, a violent acute pneumonia supervened, in others death followed from an effusion of blood into the cavity of the pleura.

In some instances, though no obvious lesion of the cardiac structure was produced, the heart has suffered so violent a commotion as to cause the suspension of its contractions, and a state of syncope which has occasionally proved fatal. The following case was not mortal, but is curious.

"A case of this description occurred while I was at Paris, and as I knew the parties concerned, and saw the patient shortly after his wound, I can vouch for its accuracy. Two French students quarrelled at supper, they wished to settle their dispute on the spot, however as they were both very tipsy and infuriated we prevented them; the next morning they met, and as they were determined that one should die, their friends prevailed on them only to load one of the pistols, and then leaving both on the table, to draw lots as to who should take the first chance of the pistols, of course being ignorant which was the loaded one; it was loaded with four pellets. They then mutually felt for the point of the chest, against which at that moment each stroke of the heart told with increased violence, and pressing firmly against this part, they fired: one of them fell to the ground in a state of insensibility, but on examing him they found merely a slight flesh wound at the part to which the pistol had been applied, and with a little care he soon came to himself. I saw him about three hours after this had occurred. He was then in a state of great anxiety which he could not account for, as he expressed more an unpleasant sensation of weight about his heart than actual pain; there was great tendency to fainting, the pulse intermitted, with severe palpitation of the heart: under proper treatment all these symptoms subsided, and he recovered perfectly in a short time. I consider myself peculiary fortunate in having witnessed this case, for in affairs of this kind it is generally the right side which is wounded, owing to the position we naturally assume; and also, it exemplifies in a striking manner the power of compressed air in resisting the expansive force of gases."

We may not be astute, but we do not exactly see our way through the preceding case. However, Mr. Lees so positively vouches for its accuracy, that scepticism must necessarily be silenced.

4. The following generalizations are not unimportant, nor are the remarks appended to them unworthy of attention.

"All parts of the heart are not equally liable to be wounded, for in a series of fifty-four cases collected by M. Olivier, the right ventricle was the seat of the wound in twenty-nine, the left ventricle in twelve, both ventricles in nine, the right auricle in three, the left in one. The same author states, that out of twenty-nine cases of penetrating wounds of the cavities of the heart, only two proved fatal within forty-eight hours; in the others death took place at the varying periods of from four to twenty-eight days after the receipt of the wound.

These differences in the time at which death has occurred in individuals whose wounds have presented nearly the same appearances, as also why they are not immediately fatal, have been attributed to the peculiar disposition of the muscular fibres of this organ; for as the heart is constituted of layers of superposed fibres having different directions, if the instrument has divided a great number of its fibres in a tranverse direction, the retraction will be considerable, and the probability of hæmorrhage much greater than if the wound ran parallel to these fibres; thus suppose the left ventricle to be wounded, there are three layers of fibres in its substance, the superficial and middle layer take one course, the deep layer takes an opposite direction, so that while one set of fibres are divided tranversely, the others are merely separated longitudinally, and thus favour the formation of a coagulum, causing an obstruction to further effusion; at the first merely temporary, but finally definitively; so that we may assume, that wounds which are parallel to the axis of the heart, are *cæteris paribus*, less rapidly fatal than those which are transverse; and it is this action of the fibres which renders wounds of the ventricles less rapidly fatal than those of the auricles."

There are some other observations to which we do not deem it necessary to advert, as our object has been to arrange and present the facts collected by the industry of Mr. Lees.

---

### Transatlantic Snuff-Chewing.

If Mrs. Trollope had given us the fol-following portrait of a fine American lady, we should have set it down at once as caricature—"and something more." Even as drawn by an American physician, who evinces a laudable antipathy to European refinements, and an ardent republican admiration of his own country, we almost hesitate to credit the fidelity of the picture!

Dr. Caleb Ticknor, in a volume on the "Philosophy of Living," which is reprinted in the American Family Library—a proof of its estimation beyond the Atlantic—favours us with the following observations on the use, or rather the abuse, of tobacco in New York.

"Could any one, entirely unacquainted with the unaccountable habits and propensities of man, after knowing the properties of tobacco, be made to believe that half the adult inhabitants of America, are passionately addicted to its use? And were he told that America's fairest daughters use it too, would he not be perfectly incredulous? And were he told, further, that ladies of the greatest respectability, the most genteel and accomplished, in one of our largest cities, carry their little jars or boxes of snuff into the social circle, and, with a delicate ivory spoon, feed their *sweet* mouths with this most delicious and agreeable poison, would he not be petrified with astonishment? How delightful it must be to have an amiable spouse rendered doubly *sweet*, and bewitchingly interesting, and most charmingly stupid and idiotic, by the constant practice of *chewing snuff;* and what a fine example to children, of the pure, cultivated taste and self-control of the mother!"

Dr. Ticknor entertains a violent antipathy to all forms and modes of using tobacco, and certainly this novel mode of taking snuff is well calculated to foster the hatred of the celebrated weed. We apprehend, however, that the smoking of a pipe or a cigar of an evening, is not so pernicious as Dr. Ticknor imagines. The practice has greatly increased since the conclusion of the late war; but it must be acknowledged that temperance in wine and spirituous liquors has also increased—and, upon the whole, the change has been for the better. We shall take another opportunity of noticing Dr. Ticknor's work, as it is not a little curious that our countryman, Mr. Mayo, has adopted the identical title of the transatlantic author, and pursued a very similar plan—the size of the two works being almost exactly the same. We have not yet had time to compare the materials of the two publications.

---

### Medicine in India.

There lie before us the numbers of the India Journal of Medical and Physical Science, for May, June, July, August, September, October, November, and December, 1836, and for January, 1837. There can, we hope, be little reason now to entertain any apprehension of its want of success. A Medical Journal is so obviously required, to bring the various members of the medical profession scattered throughout Indostan, in direct and ready communication with each other, that its advantages once felt, it will never be abandoned.

The Journal itself is conducted in a tone and with a spirit, well calculated to promote the object of its conductors and the advancement of medicine. We see in it no evidence of that truculent personality which has disgraced the pages of medical periodical literature, personality which for a time may augment the sale, but ruins the character, and ultimately destroys the influence of the journal in which it is permitted. Mr. Corbyn, the editor of the India Journal, feels what is due to himself as a gentleman, to his profession as a science, and to all medical men as his peers, and both his good feeling and sound sense, will tell him that their interests and their honour are his own.

The majority of the original communications in the India Journal, are on diseases which either so exclusively affect the residents in hot climates, or on those which are so much modified by high temperature and locality, as not to interest readers in this country. To such papers we will not refer, but simply cull a fact or an observation here and there from the pages of our valuable contemporary.

I. *Operation for removing the Deformity succeeding Burns.*

Mr. Chapman, of Purneah, has addressed a short communication on the best mode of operating for the removal of deformities succeeding burns of the arm and fore-arm. The following extract will explain all that is necessary.

"The appearance which this deformity presents is, I believe, always the same,—a thin but deep bridle of integument connecting the arm and fore-arm, and forming at the elbow an angle more or less acute.—In performing the necessary operation, little more seems to be required than a simple incision dividing the integuments fairly to the bend of the arm, and keeping up proper and continued extension,—such is the opinion of Dupuytren and others.—Performing this operation by a single incision, cannot, I am persuaded, remedy this deformity to any great extent, as it will be almost impossible to prevent the cicatrix from again contracting as the wound heals. In one instance this took place in a patient under my care; I, however, subsequently adopted the operation I shall now detail; and which will be found to answer better than either of those proposed by Dupuytren or Mr. Roberts. It consists in making two incisions, so close to the arm and fore-arm as to include a portion of the normal integuments of both. An incision of this description, giving to the excised part a triangular form; a good deal of blood necessarily flows, but it is easily restrained by pressure. The advantage of this mode appears to be, that the arm is brought immediately to nearly its natural form, and the wound, while the healing process advances, is much less disposed to contract. Lint smeared with a little simple ointment to cover the raw surfaces is now used, and two fracture splints, on the arm and fore-arm, are to be applied with moderate pressure. I now come to that portion of the treatment on which depends the success of the operation. This consists in keeping up a proper degree of extension by suspending from the wrist a weight, of at first ℔iss, and increasing the weight as the cure proceeds; at the same time binding at the wrist and top of the shoulder an elastic piece of bamboo band, drawing with a bandage the centre of it towards the bend of the arm; this is of much consequence, as it forms an antagonist power to the flexors, which the patient, to receive temporary relief from the suspended weight, constantly throws into action."

II. *Hydrocele Treated by Injection with Iodine.*

Case. "John M'Evoy, æt. 40: a man of sanguineous and nervous temperament, with light hair, blue eyes, and very fair complexion, admitted with fever, disordered bowels, and chronic hepatitis. He remained under treatment for these complaints till the 8th of December, when he was pretty well.

He showed me that day a hydrocele of the right side. The testicle could be felt distinctly somewhat larger than the opposite, and flattened, the epididemis swollen. The fluid began to collect about twelve months ago, and was the consequence of an inflammation of the testicle caused by his accidentally hitting himself with his own hand while playing at racketts. He had been leeched repeatedly and confined to bed for some weeks, but the testicle always continued a little painful. He now suffers no inconvenience except from the size of the tumor, and is anxious to be relieved—2lb. of a limpid fluid were drawn off by the trocar, and the emptied sac immediately injected with tinct. iodine ʒss., aqua distil. ʒiij., when the trocar was withdrawn.

Contrary to my expectation he did not complain of much pain at the time. The following day, there was active inflammation, the scrotum as large as before the operation, pain described as that of ant's stinging along the under surface of the scrotum, and along the spermatic chord, no constitutional fever; bowels regular.

A cold saturnine lotion was directed and a dose of salts."

On the 22d, the testicles were of the same size, and he was discharged. The author of the case is very sanguine on the effects of the injection of iodine. He declares that out of 400 cases not one has failed. He think this method will supersede all others. Perhaps it may, but we are not sure of it. No one method is adapted for all cases.

III. *On Wounds incidental to Artillerymen, from the sudden Explosion of the Cartilage in loading.*

The following communication from Mr. Baddeley deserves the consideration of our naval and military brethren. The accident to which it refers is not uncommon.

" In accidents of this nature, two men usually suffer, the Loadsman and the Ventsman. In that occurring to the former, there is always considerable injury done to the forearm, the flesh of which is torn to rags, and the bones and hand shattered to pieces. It is also occasionally complicated with a fracture of the humerus, amputation of the upper third of the fore-arm is generally called for (the humerus if broken, having been previously set,) and when performed soon after the infliction of the injury, no bad symptom need be apprehended.

The muscles on being cut, present a dark and discoloured appearance, as if severely bruised; and the surface of the stumps is irregular, owing to a loss of the contractile power in the fibre; for which reason also, the bones should be sawn higher than usual.

Minute splinters of the sponge staff and bits of cloth are apt to be forced into the substance of the integuments, and may cause considerable constitutional disturbance, which is much relieved by blood-letting and the application of cold water.

In that occurring to the Ventsman, the pad of the thumb may be ripped up, or the 2d phalanx entirely blown off, exposing the articulating process of the first, which sometimes requires to be sawn off, to admit of the lacerated integuments covering the extremity of the four, here the pain and inflammation is often considerable; the repeated application, therefore, of leeches and cold water, indicated.

In consequence of the number of such accidents, I think that the means of obviating their recurrence, may be considered a subject worthy of consideration.

It is customary to attribute their occurrence to the imperfect manner in which the vent has been served; in my opinion, and in that of many artillery officers, erroneously; for, in five cases I have been witness to, most of the ventsmen were invalided in consequence of the injury done to their hands, and in two instances, the 2d phalanx was entirely blown off, clearly demonstrating the perfect manner in which the vent was served, and also the impossibility of preventing such accidents by this method.

We are hence induced to inquire in another direction, for the true cause of this serious occurrence, and are naturally led to consider the state of the cartridge itself. The *surge* or cloth used, is of so *open* a texture, as to permit the fine meal powder to penetrate, and in many instances to *cover* the outside of the cartridge, particularly after it has received a violent shaking in the carriage.

From this fact, it is easily conceivable, how instantaneously the slightest spark remaining in the chamber of the gun, could communicate with, and inflame, the whole.

It is observable too, that the accident rarely if ever occurs in battery, where the cartridges are made up as required, and suffer no concussion from carriage. The remedy then is only to be expected from using cloth of a more compact texture, or by steeping it in some composition that will fill up the interstices, without diminishing its pliancy. Should it not however be deemed advisable to adopt this means of preventing the occurrence altogether, the next point to be considered is whether some method may not be devised for preventing injury to the man who serves the vent:

For instance, instead of his thumb might not a *pad* be used, somewhat similar to printer's ink dabber, covered with sheep skin? This would answer two purposes; one to close the vent securely against fire from outside, the other as a substitute for the piece of tow now in use, for brushing away the dirt from the breech. And here, permit me to observe upon the custom of invaliding artillerymen, for the loss or injury merely of their *left* thumbs, which has been supposed to render them unfit for the performance of their duties in that service:

It is notorious that they are *still capable* of performing every duty connected with that branch, with the single exception of serving the vent with the thumb of their left hands. (However, were the pad used they would still be effective even for that duty.)

Those who advocate the system, can but adduce an extreme case, where two men only are left to serve the gun; but here, one must act as loadsman, while the other primes and fires; and should he feel disposed to serve the vent at all, he does so with his *right* thumb; for he stands on the *left of the trail,* as matter of course, so that there is *no reason* for considering such a man inefficient."

IV. *Mode of treating a severe Wound of the Tongue.*

This case is reported by Mr. Jackson, surgeon to the 8th Light Cavalry.

*Case.* " J. L. J., a lively active child, was very busy on the morning of the 15th of August assisting the bearers to air the clothes, furniture, &c. &c. He took up a book and was running with it towards the house, when his foot slipped, on a green patch caused by the rains, near the pukka step, and his chin coming against it, his tongue, which must have been partly cut at the time, was forced with great violence against the upper teeth (which were loosened by the concussion) and almost cut through its anterior third, opposite the frænum. The wound, being of a contused as well as of an incised kind, did not bleed much, but on desiring the little patient to put out the tongue for the purpose of examining it, the anterior part fell quite over the lip, and the edges of the wound receded so much, that the end of the little finger might have been introduced into the cleft.

This effect must have been produced by an irregular action of the anterior fibres of the genio-hyo-glossus muscle, which were no longer counteracted by the lingualis. However that may be, I was quite satisfied with a short inspection, and directed the boy to draw the tongue within the mouth, keeping it open for a few seconds, that I might see the effects.

I was delighted to find that, in this situation, the flap, if I may be allowed to call it so, exactly filled up the hollow space, and the only thing to be done was to keep the parts in apposition, and do as little as possible, till union took place, and nature completed her own cure.

I therefore bandaged up the lower jaw to keep all quiet, told my little restless but sensible patient that he must on no account speak; and having procured a swan quill, I cut it into a proper shape for him to suck liquid through it. This was rather troublesome, as on his first attempt he succeeded in blowing the victuals in the contraiy direction to his mouth, but by a little patience, he was soon taught enough of pneumatics to make a vacuum with his lips and thought it excellent fun. On the following morning, I ventured to look at the wound; with fear and trembling, which I did, by telling the boy to open his mouth without making any other kind of motion, and was glad to find that the wound looked well. On the third day it had quite closed, leaving a red bloody line, and in a week nothing was discernible except a line not thicker than a hair. For several days I enjoined silence to keep the tongue as quiet as I could, and in so young a child, this was a matter of no small difficulty; with the temptation of bearers and servants of all descriptions trying to amuse him. During the first twenty-four hours, however, I may say he scarcely uttered a syllable and expressed assent and dissent by motions of his head."

The usual directions for treating this accident lean too exclusively on the use of the suture. This case shews the propriety of an intelligent surgeon's always using his own eyes, and exercising his own judgment. It may convey a hint to other surgeons whose eyes and judgment are not always available.

---

### REMARKABLE CASE OF CEPHALÆA.

Mr. Jeffreys, whom we know to be an intelligent surgeon of Wisbeach, has related an instructive case in our contemporary, the Medical Gazette, of which the following is a condensed account.

E. Eldridge, aged 19 years, a seafaring man, was condemned to the tread-mill, in the Elm work-house, where he worked from the 20th January till the 12th February, 1837, when Mr. J. saw him. He then complained of pain in the fore part of the head, occasionally remitting. There was some quickness of pulse, and whiteness of tongue, but no other febrile symptom. He was excused from duty for a week, when, being suspected of skulking, he was again remanded to the tread-mill on the 20th. On the 22d this man still persisted that whenever he got up, the pain in his head recurred, or was aggravated. He was now closely questioned, and it was found that he had similar head-aches on board H. M. ship Dreadnought, about a year previously. It was now believed that his complaints were not fictitious, though there was little appearance of any serious illness. None of the senses were affected, the bowels were regular—the appetite unimpaired, and the secretions natural, pulse 96, tongue rather furred, complexion ruddy.

April 1. The pain increased, and was more steady in the back part of the head, much aggravated by raising the head from the pillow, and the patient was evidently emaciating. The opinion of Dr. Whitsed was now requested, and it was noted that the pulse was 120, rather small—pupils dilated—tongue white—complexion sallow—body attenuated—much pain on raising the head—intellect and motive powers unimpaired. The head was shaved, and a large blister applied to the scalp. Antimony and digitalis.—12th. Slept well last night; but died suddenly this afternoon, as was believed in a convulsion.

On examination, the vessels of the dura mater appeared turgid. The inner meninges were also injected; but there was no infiltration. In the lateral ventricles there were found two ounces of clear fluid. The plexus choroides and lining of the ventricles were very vascular. On removing the tentorium, the left lobe of the cerebellum appeared to be much larger than the right, and on cutting into it a globular tubercle, an inch in diameter, was divided, it being of firmer consistence than the brain, but unorganized. The substance of the cerebellum around the tubercle was softened to the consistance of custard. In the centre of the tubercle was a minute cavity, looking as if it had originally contained a small coagulum. Time did not permit to examine the thorax or abdomen; but it is not likely that any organic disease would have been there discovered.

This is one of those instances (by no means rare) where we are at a loss to account for the paucity of symptoms during life, and the sudden occurrence of death!

